# Supplementary material for: Novel Indane Derivatives with Antioxidant Activity from the Roots of Anisodus tanguticus
Source: Molecules. 2023 Feb 3;28(3):1493. doi: 10.3390/molecules28031493 (PMC9919654; doi:10.3390/molecules28031493)
Supplement: Supplementary file 1 [file molecules-28-01493-s001.zip › molecules-2169740-supplementary.pdf]

Supplementary material

Novel indane derivatives with antioxidant activity from the roots of *Anisodus tanguticus*

Chun-Wang Meng [1,2,†], Hao-Yu Zhao [1,2,†], Huan Zhu [1,2], Cheng Peng [1], Qin-Mei Zhou [1,2,3,*] and Liang Xiong [1,2,*]

1 State Key Laboratory of Southwestern Chinese Medicine Resources, School of Pharmacy, Chengdu University of Traditional Chinese Medicine, Chengdu 611137, China
2 Institute of Innovative Medicine Ingredients of Southwest Specialty Medicinal Materials, School of Pharmacy, Chengdu University of Traditional Chinese Medicine, Chengdu 611137, China
3 Innovative Institute of Chinese Medicine and Pharmacy, Chengdu University of Traditional Chinese Medicine, Chengdu 611137, China
* Correspondence: zhqmyx@sina.cn (Q.-M.Z); xiling@cdutcm.edu.cn (L.X.)
† These authors contributed equally to this work.

The List of Contents

No.	Content	Page
1	**Text S1. ECD Calculation of Compound 2**	S1
2	**Figure S1.** *ω*B97XD/DGDZVP Optimized 18 Conformers of (2*S*,2′*S*)-**2**	S1
3	**Table S1.** Energy Analysis for the Conformers of (2*S*,2′*S*)-**2**	S2
4	**Text S2. ECD Calculation of Compound 4**	S3
5	**Figure S2.** *ω*B97XD/DGDZVP Optimized 11 Conformers of (3*R*,3a*S*,8b*S*)-**4**	S4
6	**Table S2.** Energy Analysis for the Conformers of (3*R*,3a*S*,8b*S*)-**4**	S4
7	**Figure S3.** The UV spectrum of compound **1** in MeCN	S6
8	**Figure S4.** The IR Spectrum of Compound **1**	S7
9	**Figure S5.** The (+)-HR-ESI-MS Spectroscopic Data of Compound **1**	S8
10	**Figure S6.** The ^{1}H NMR Spectrum of Compound **1** in Acetone-d_6	S9
11	**Figure S7.** The ^{13}C NMR Spectrum of Compound **1** in Acetone-d_6	S10
12	**Figure S8.** The DEPT Spectrum of Compound **1** in Acetone-d_6	S11
13	**Figure S9.** The HSQC Spectrum of Compound **1** in Acetone-d_6	S12
14	**Figure S10.** The ^{1}H-^{1}H COSY Spectrum of Compound **1** in Acetone-d_6	S13
15	**Figure S11.** The HMBC Spectrum of Compound **1** in Acetone-d_6	S14
16	**Figure S12.** The UV spectrum of compound **2** in MeCN	S15
17	**Figure S13.** The IR Spectrum of Compound **2**	S16
18	**Figure S14.** The (+)-HR-ESI-MS Spectroscopic Data of Compound **2**	S17
19	**Figure S15.** The ^{1}H NMR Spectrum of Compound **2** in Acetone-d_6	S18
20	**Figure S16.** The ^{13}C NMR Spectrum of Compound **2** in Acetone-d_6	S19
21	**Figure S17.** The DEPT Spectrum of Compound **2** in Acetone-d_6	S20
22	**Figure S18.** The HSQC Spectrum of Compound **2** in Acetone-d_6	S21
23	**Figure S19.** The ^{1}H-^{1}H gCOSY Spectrum of Compound **2** in Acetone-d_6	S22
24	**Figure S20.** The HMBC Spectrum of Compound **2** in Acetone-d_6	S23
25	**Figure S21.** The UV spectrum of compound **3** in MeCN	S24
26	**Figure S22.** The IR Spectrum of Compound **3**	S25
27	**Figure S23.** The (+)-HR-ESI-MS Spectroscopic Data of Compound **3**	S26
28	**Figure S24.** The ^{1}H NMR Spectrum of Compound **3** in Acetone-d_6	S27
29	**Figure S25.** The ^{13}C NMR Spectrum of Compound **3** in Acetone-d_6	S28
30	**Figure S26.** The DEPT Spectrum of Compound **3** in Acetone-d_6	S29
31	**Figure S27.** The edit-HSQC Spectrum of Compound **3** in Acetone-d_6	S30
32	**Figure S28.** The ^{1}H-^{1}H COSY Spectrum of Compound **3** in Acetone-d_6	S31
33	**Figure S29.** The HMBC Spectrum of Compound **3** in Acetone-d_6	S32
34	**Figure S30.** The 1D NOE Spectrum of Compound **3** in Acetone-d_6	S33
35	**Figure S31.** The UV spectrum of compound **4** in MeCN	S34
36	**Figure S32.** The IR Spectrum of Compound **4**	S35

37	**Figure S33.** The (+)-HR-ESI-MS Spectroscopic Data of Compound **4**	S36
38	**Figure S34.** The ^{1}H NMR Spectrum of Compound **4** in Acetone-d_6	S37
39	**Figure S35.** The ^{13}C NMR Spectrum of Compound **4** in Acetone-d_6	S38
40	**Figure S36.** The DEPT Spectrum of Compound **4** in Acetone-d_6	S39
41	**Figure S37.** The edit-HSQC Spectrum of Compound **4** in Acetone-d_6	S40
42	**Figure S38.** The ^{1}H-^{1}H COSY Spectrum of Compound **4** in Acetone-d_6	S41
43	**Figure S39.** The HMBC Spectrum of Compound **4** in Acetone-d_6	S42
44	**Figure S40.** The 1D NOE Spectrum of Compound **4** in Acetone-d_6	S43

Text S1. ECD Calculation of Compound 2.

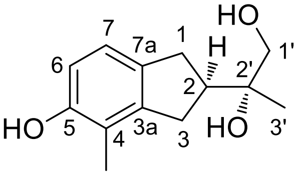

(2*R*,2′*R*)-**2**

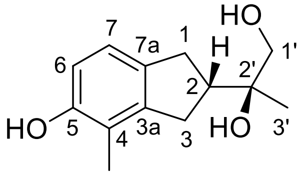

(2*S*,2′*S*)-**2**

Conformation searches based on molecular mechanics with MMFF94s force field were performed for (2*S*,2′*S*)-**2** and gave 18 conformers (Boltzmann distribution ≥ 1%, Figure S1) [32,33]. The selected conformers were optimized using DFT at B3LYP/6-31G (d) level in vacuum with the Gaussian 16 program (Table S1) [34]. The B3LYP/6-31G (d)-optimized conformers (Boltzmann distribution ≥1%) were then reoptimized at the *ω*B97XD/DGDZVP level in acetonitrile. ECD computations for the *ω*B97XD/DGDZVP-optimized conformers (Boltzmann distribution ≥1%; Figure S1) were carried out at the CAM-B3LYP/DGDZVP level in acetonitrile [35]. Finally, according to the Boltzmann distribution theory and their relative Gibbs free energy (ΔG), the ECD spectrum for (2*S*,2′*S*)-**2** was generated using SpecDis 1.71 with σ = 0.25 eV and a UV shift of +13 nm [36]. The corresponding theoretically ECD spectrum of (2*R*,2′*R*)-**2** was depicted by inverting that of (2*S*,2′*S*)-**2**. In the region of 190–300 nm, the theoretically calculated ECD spectrum of (2*R*,2′*R*)-**2** was in agreement with the experimental ECD spectrum of **2**.

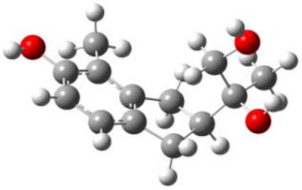

(2*S*,2′*S*)-**2**-C1

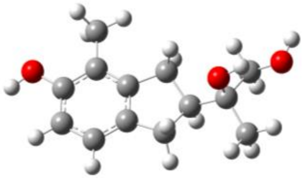

(2*S*,2′*S*)-**2**-C2

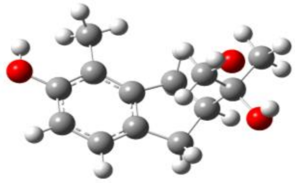

(2*S*,2′*S*)-**2**-C3

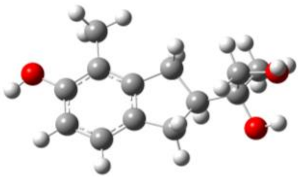

(2*S*,2′*S*)-**2**-C4

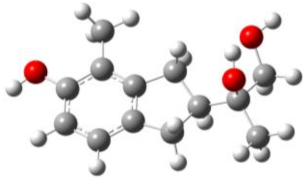

(2*S*,2′*S*)-**2**-C5

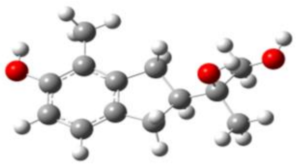

(2*S*,2′*S*)-**2**-C6

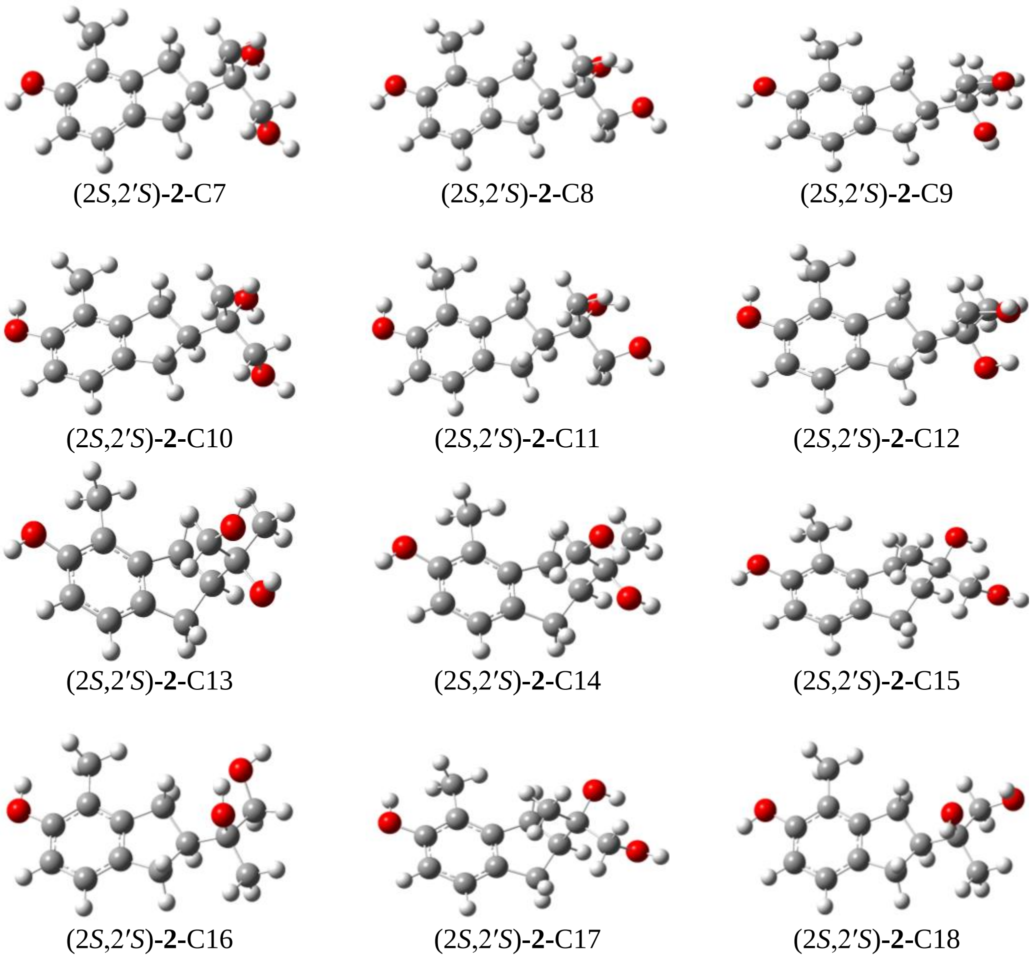

Figure S1. *ω*B97XD/DGDZVP Optimized 18 Conformers of (2*S*,2′*S*)-**2** (Boltzmann distribution ≥1%).

Table S1. Energy Analysis for the Conformers of (2*S*,2′*S*)-**2**.

Conf.	MMFF energy	B3LYP/6-31G(d) Gibbs free energy (298.15 K)			*ω*B97XD/DGDZVP Gibbs free energy (298.15 K)		
	ΔE (Kcal/mol)	*G* (Hartree)	*ΔG* (Kcal/mol)	Boltzmann Distribution	*G* (Hartree)	*ΔG* (Kcal/mol)	Boltzmann Distribution
(2*S*,2′*S*)-**2**-C1	0.0000	-731.631043	0.0000	0.010	-731.503033	0.0000	0.022
(2*S*,2′*S*)-**2**-C2	0.7631	-731.633726	-1.6840	0.173	-731.504547	-0.9500	0.110
(2*S*,2′*S*)-**2**-C3	0.7706	-731.629808	0.7750	0.003	-731.502074	0.6020	0.008
(2*S*,2′*S*)-**2**-C4	1.0133	-731.631526	-0.3030	0.017	-731.50302	0.0080	0.022
(2*S*,2′*S*)-**2**-C5	1.3916	-731.632504	-0.9170	0.047	-731.503556	-0.3280	0.039

(2*S*,2′*S*)-**2**-C6	1.3958	-731.632445	-0.8800	0.045	-731.503757	-0.4540	0.048
(2*S*,2′*S*)-**2**-C7	1.4891	-731.632646	-1.0060	0.055	-731.504778	-1.0950	0.141
(2*S*,2′*S*)-**2**-C8	1.5820	-731.632155	-0.6980	0.033	-731.503888	-0.5370	0.055
(2*S*,2′*S*)-**2**-C9	1.6115	-731.632538	-0.9380	0.049	-731.503697	-0.4170	0.045
(2*S*,2′*S*)-**2**-C10	1.6928	-731.631623	-0.3640	0.019	-731.503695	-0.4150	0.045
(2*S*,2′*S*)-**2**-C11	1.7104	-731.631189	-0.0920	0.012	-731.502748	0.1790	0.016
(2*S*,2′*S*)-**2**-C12	1.7298	-731.630251	0.4970	0.004	-731.501551	0.9300	0.005
(2*S*,2′*S*)-**2**-C13	1.7839	-731.631089	-0.0290	0.011	-731.502772	0.1640	0.017
(2*S*,2′*S*)-**2**-C14	1.8334	-731.631657	-0.3850	0.019	-731.503819	-0.4930	0.051
(2*S*,2′*S*)-**2**-C15	1.8721	-731.63169	-0.4060	0.020	-731.504428	-0.8750	0.097
(2*S*,2′*S*)-**2**-C16	1.8844	-731.631306	-0.1650	0.013	-731.502833	0.1260	0.018
(2*S*,2′*S*)-**2**-C17	1.9952	-731.630702	0.2140	0.007	-731.503127	-0.0590	0.025
(2*S*,2′*S*)-**2**-C18	2.0082	-731.634654	-2.2660	0.463	-731.505269	-1.4030	0.237

Text S2. ECD Calculation of Compound 4.

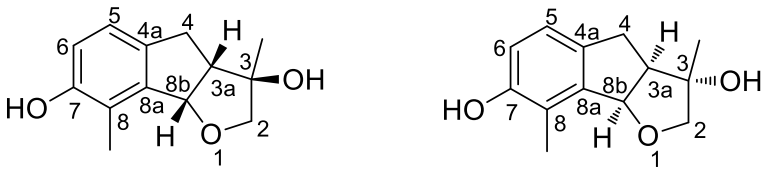

(3*R*,3a*S*,8b*S*)-**4** (3*S*,3a*R*,8b*R*)-**4**

Conformation searches based on molecular mechanics with MMFF94s force field were performed for (3*R*,3a*S*,8b*S*)-**4** and gave 11 conformers [32,33]. The selected conformers were optimized using DFT at B3LYP/6-31G (d) level in vacuum with the Gaussian 16 program (Table S2) [34]. The B3LYP/6-31G (d)-optimized conformers (Boltzmann distribution ≥ 1%) were then reoptimized at the *ω*B97XD/DGDZVP level in acetonitrile. ECD computations for the *ω*B97XD/DGDZVP-optimized conformers (Boltzmann distribution ≥ 1%; Figure S2) were carried out at the CAM-B3LYP/DGDZVP level in acetonitrile [35]. Finally, according to the Boltzmann distribution theory and their relative Gibbs

free energy (ΔG), the ECD spectrum for (3*R*,3a*S*,8b*S*)-**4** was generated using SpecDis 1.71 with σ = 0.25 eV and a UV shift of +15 nm [36]. The corresponding theoretical ECD spectrum of (3*S*,3a*R*,8b*R*)-**4** was depicted by inverting that of (3*R*,3a*S*,8b*S*)-**4**. The theoretically calculated ECD spectrum of (3*S*,3a*R*,8b*R*)-**4** was in agreement with the experimental ECD spectrum of **4**.

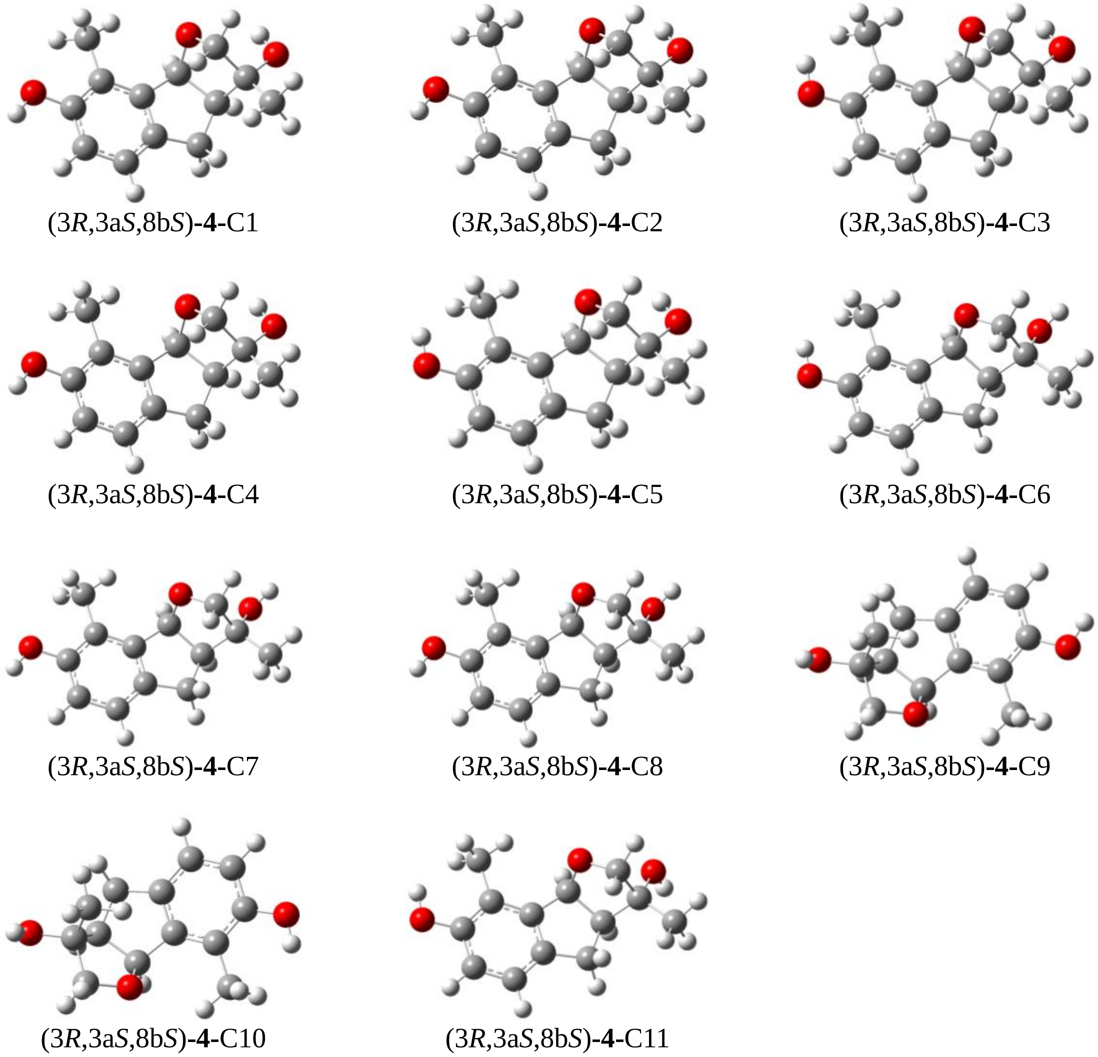

Figure S2. *ω*B97XD/DGDZVP Optimized 11 Conformers of (3*R*,3a*S*,8b*S*)-**4** (Boltzmann distribution ≥1%).

Table S2. Energy Analysis for the Conformers of (3*R*,3a*S*,8b*S*)-**4**.

Conf.	MMFF energy	B3LYP/6-31G(d) Gibbs free energy (298.15 K)			*ω*B97XD/DGDZVP Gibbs free energy (298.15 K)		
	ΔE	*G*	*ΔG*	Boltzmann	*G*	*ΔG*	Boltzmann

	(Kcal/mol)	(Hartree)	(Kcal/mol)	Distribution	(Hartree)	(Kcal/mol)	Distribution
(3*R*,3a*S*,8b*S*)-**4**-C1	0.0000	-730.447328	0.0000	0.227	-730.312657	0.0000	0.196
(3*R*,3a*S*,8b*S*)-**4**-C2	0.0056	-730.447328	0.0000	0.227	-730.312661	-0.0030	0.196
(3*R*,3a*S*,8b*S*)-**4**-C3	0.1000	-730.446837	0.3080	0.135	-730.311746	0.5720	0.075
(3*R*,3a*S*,8b*S*)-**4**-C4	0.7462	-730.447328	0.0000	0.227	-730.312664	-0.0040	0.197
(3*R*,3a*S*,8b*S*)-**4**-C5	0.8230	-730.446835	0.3090	0.135	-730.311755	0.5660	0.075
(3*R*,3a*S*,8b*S*)-**4**-C6	0.9569	-730.444569	1.7310	0.012	-730.311104	0.9750	0.038
(3*R*,3a*S*,8b*S*)-**4**-C7	1.0530	-730.444755	1.6150	0.015	-730.311916	0.4650	0.089
(3*R*,3a*S*,8b*S*)-**4**-C8	1.1136	-730.444758	1.6130	0.015	-730.311916	0.4650	0.089
(3*R*,3a*S*,8b*S*)-**4**-C9	1.6685	-730.442817	2.8310	0.002	-730.309812	1.7850	0.010
(3*R*,3a*S*,8b*S*)-**4**-C10	1.6758	-730.44256	2.9920	0.001	-730.309139	2.2080	0.005
(3*R*,3a*S*,8b*S*)-**4**-C11	1.7977	-730.443671	2.2950	0.005	-730.310904	1.1000	0.031

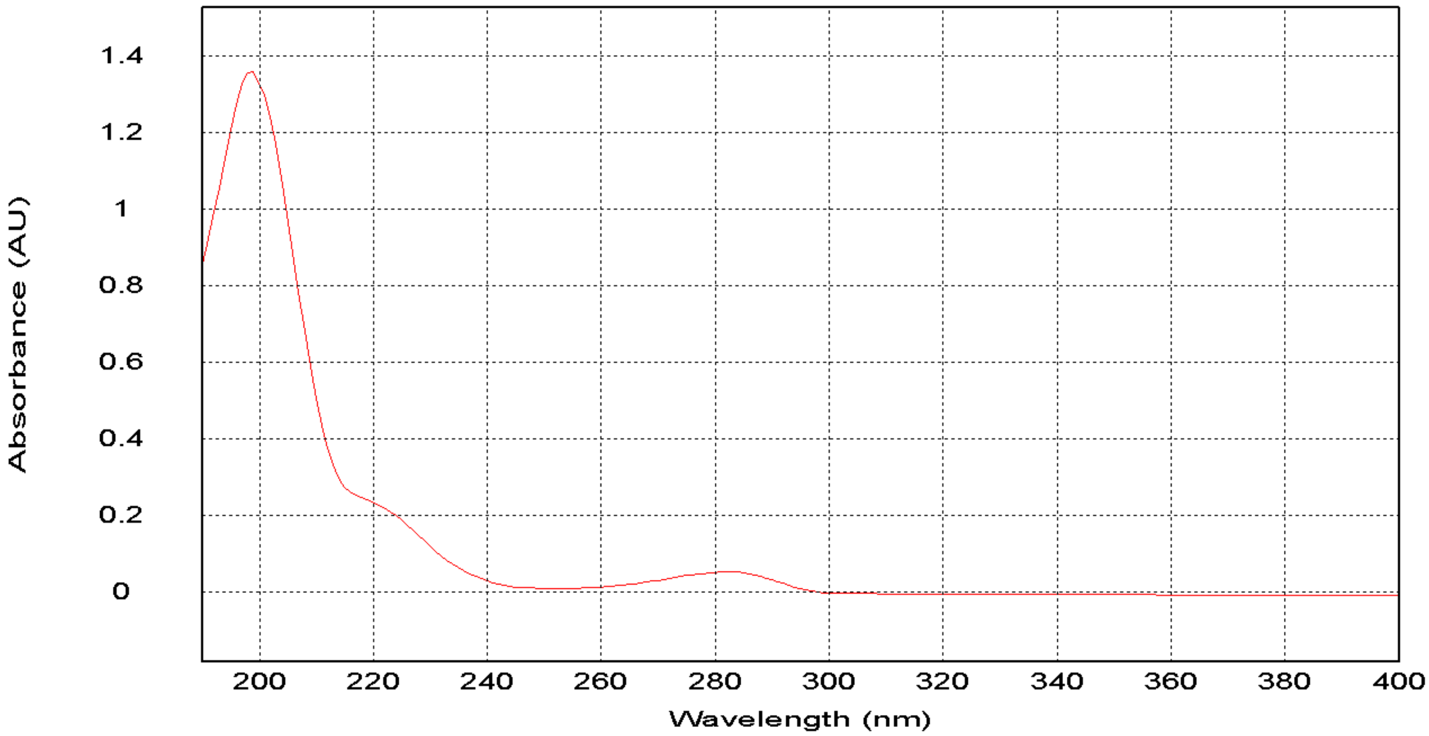

Figure S3. The UV spectrum of compound **1** in MeCN.

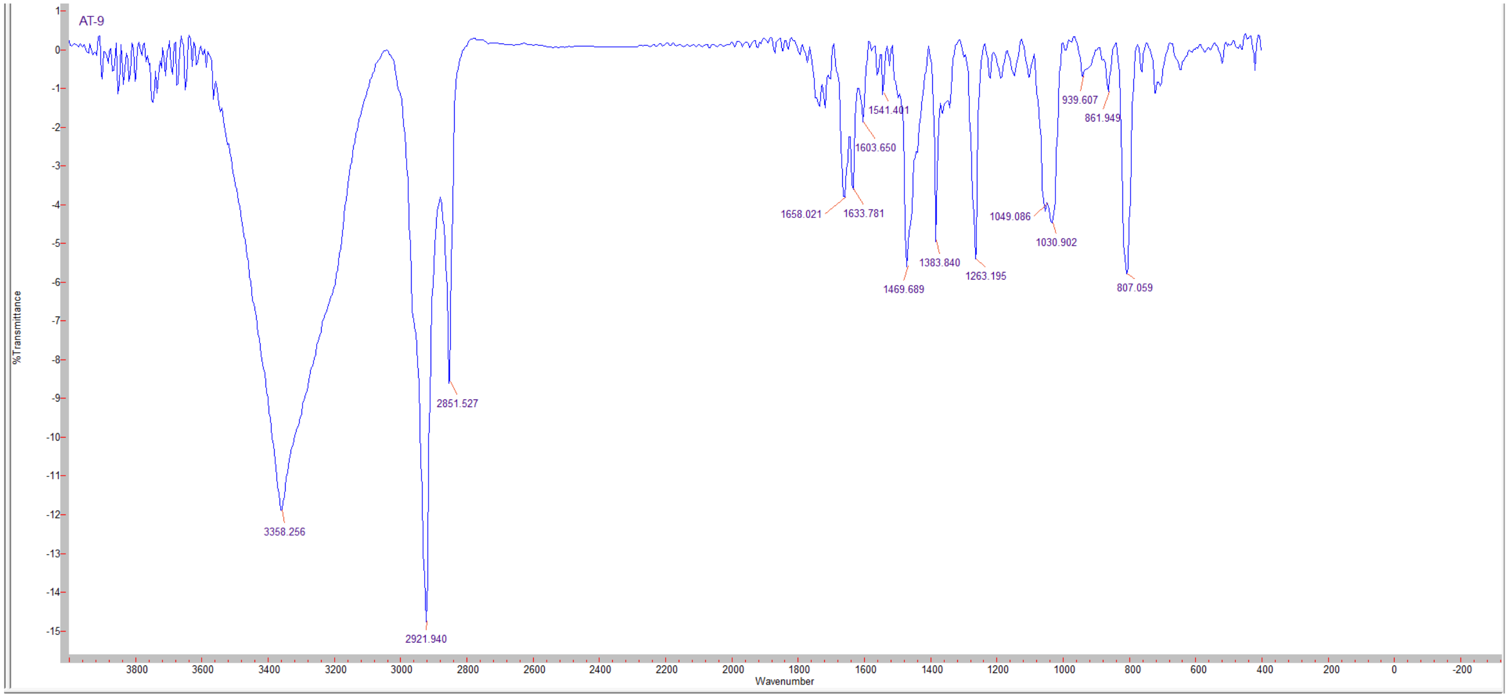

Figure S4. The IR Spectrum of Compound **1**.

Single Mass Analysis
Tolerance = 0.6 mDa / DBE: min = -1.5, max = 50.0
Element prediction: Off
Number of isotope peaks used for i-FIT = 3
Monoisotopic Mass, Even Electron Ions
70 formula(e) evaluated with 1 results within limits (up to 50 best isotopic matches for each mass)
Elements Used:

Mass	Calc. Mass	mDa	PPM	DBE	Formula	i-FIT	i-FIT Norm	Fit Conf %	C	H	O	Na
245.1157	245.1154	0.3	1.2	4.5	C13 H18 O3 Na	127.6	n/a	n/a	13	18	3	1

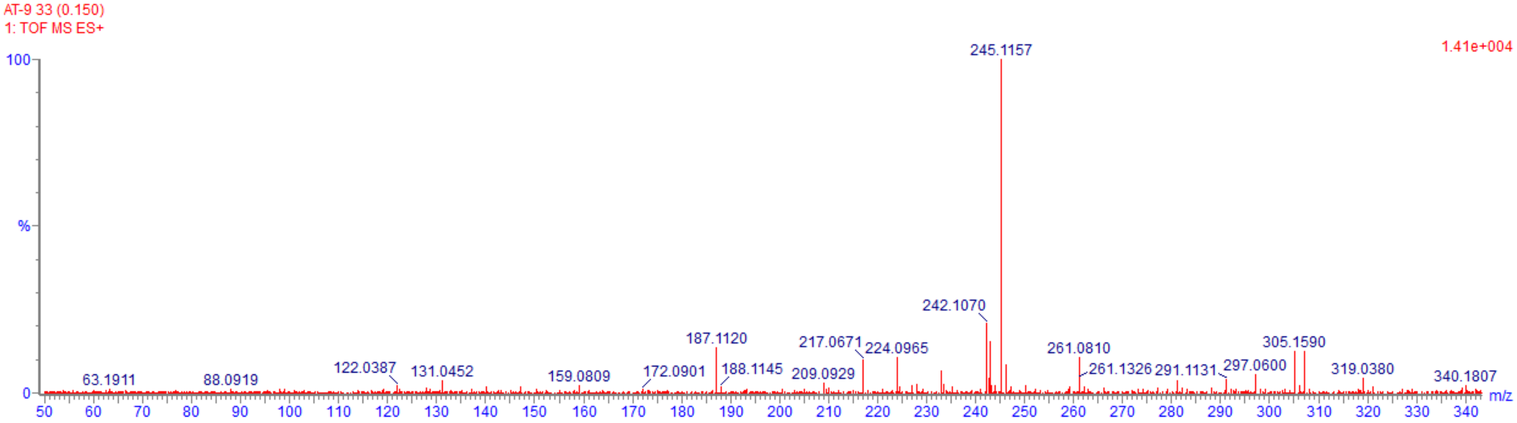

Figure S5. The (+)-HR-ESI-MS Spectroscopic Data of Compound **1**.

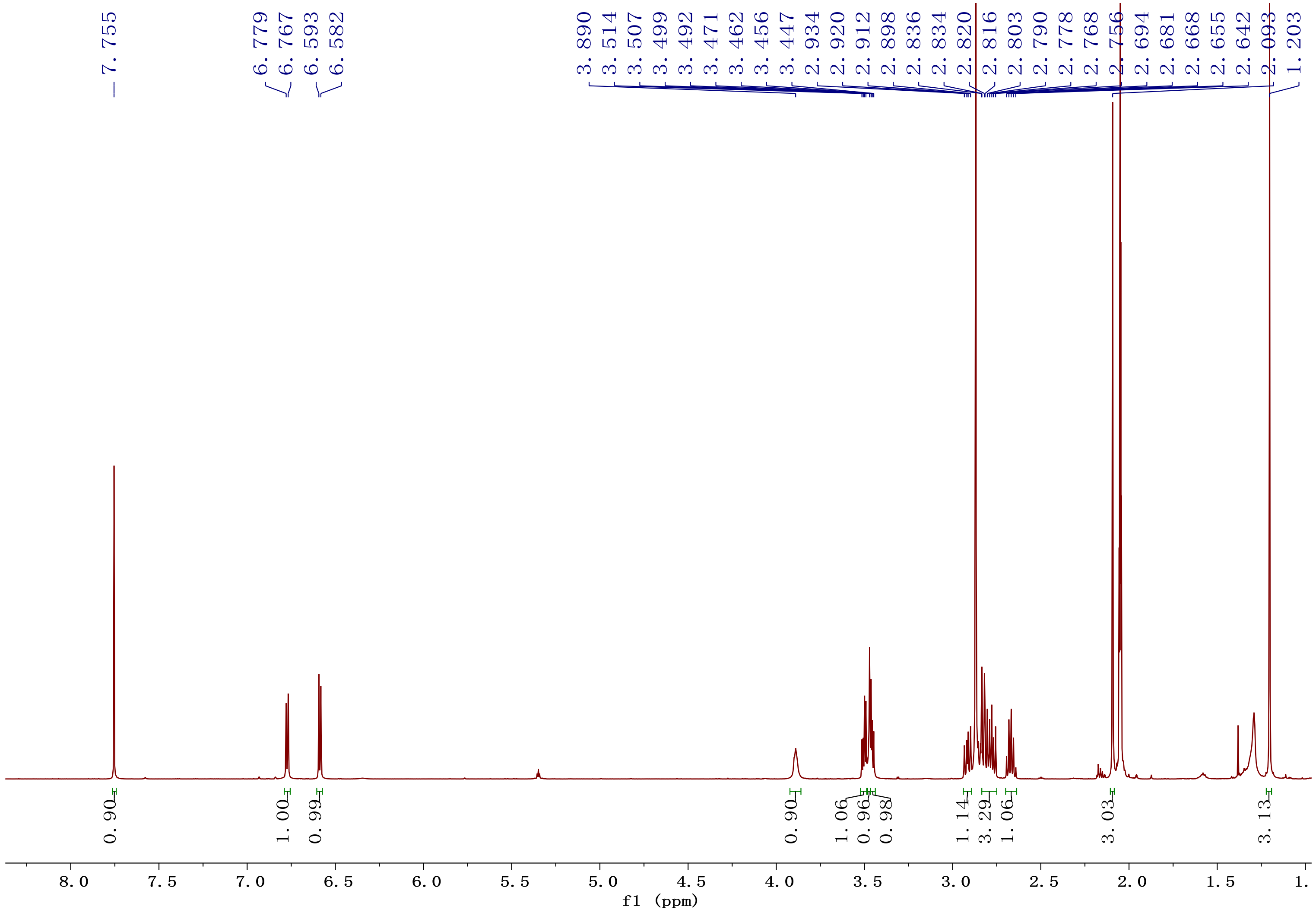

Figure S6. The ^{1}H NMR Spectrum of Compound **1** in Acetone-d_6.

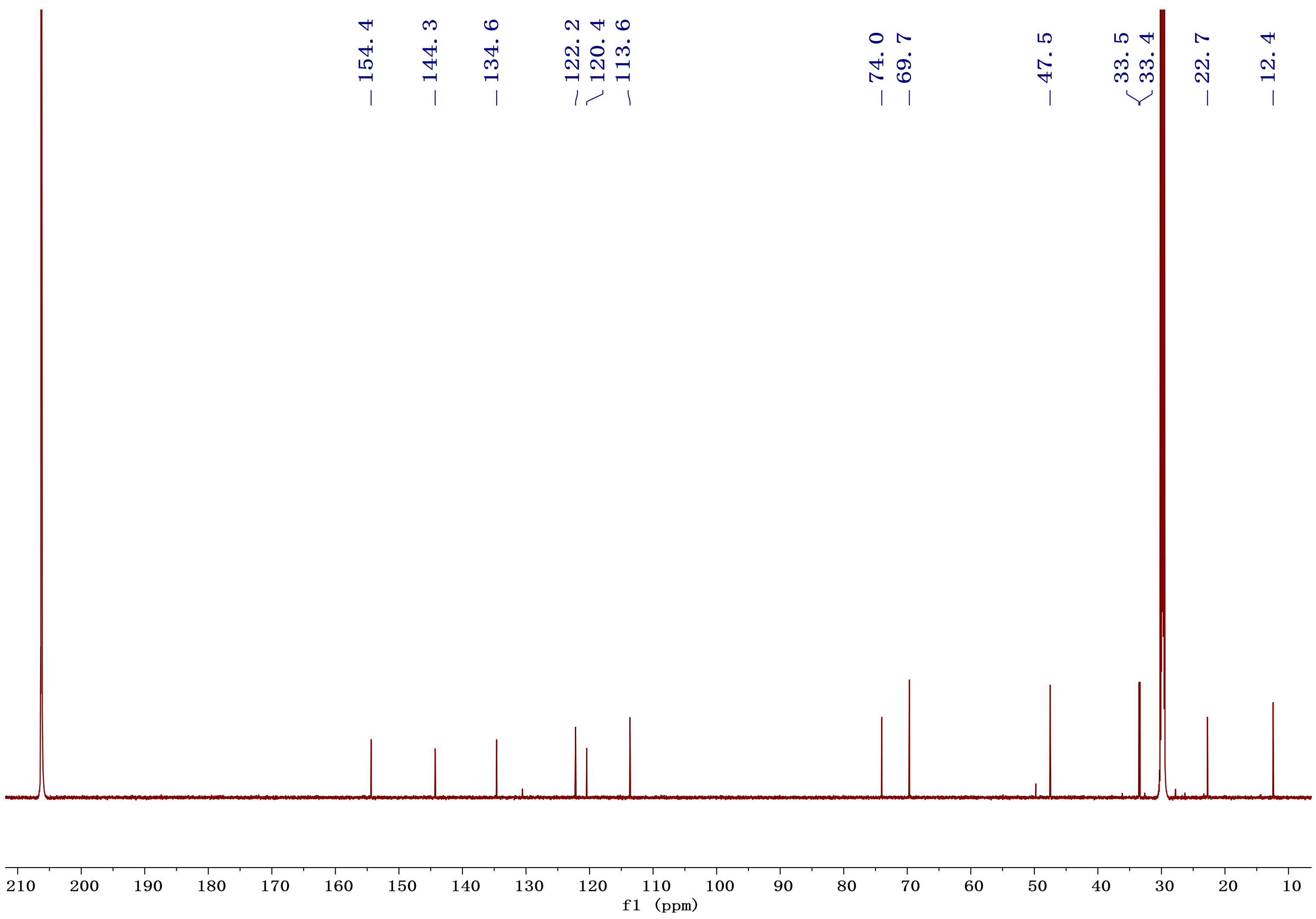

Figure S7. The ^{13}C NMR Spectrum of Compound **1** in Acetone-d_6.

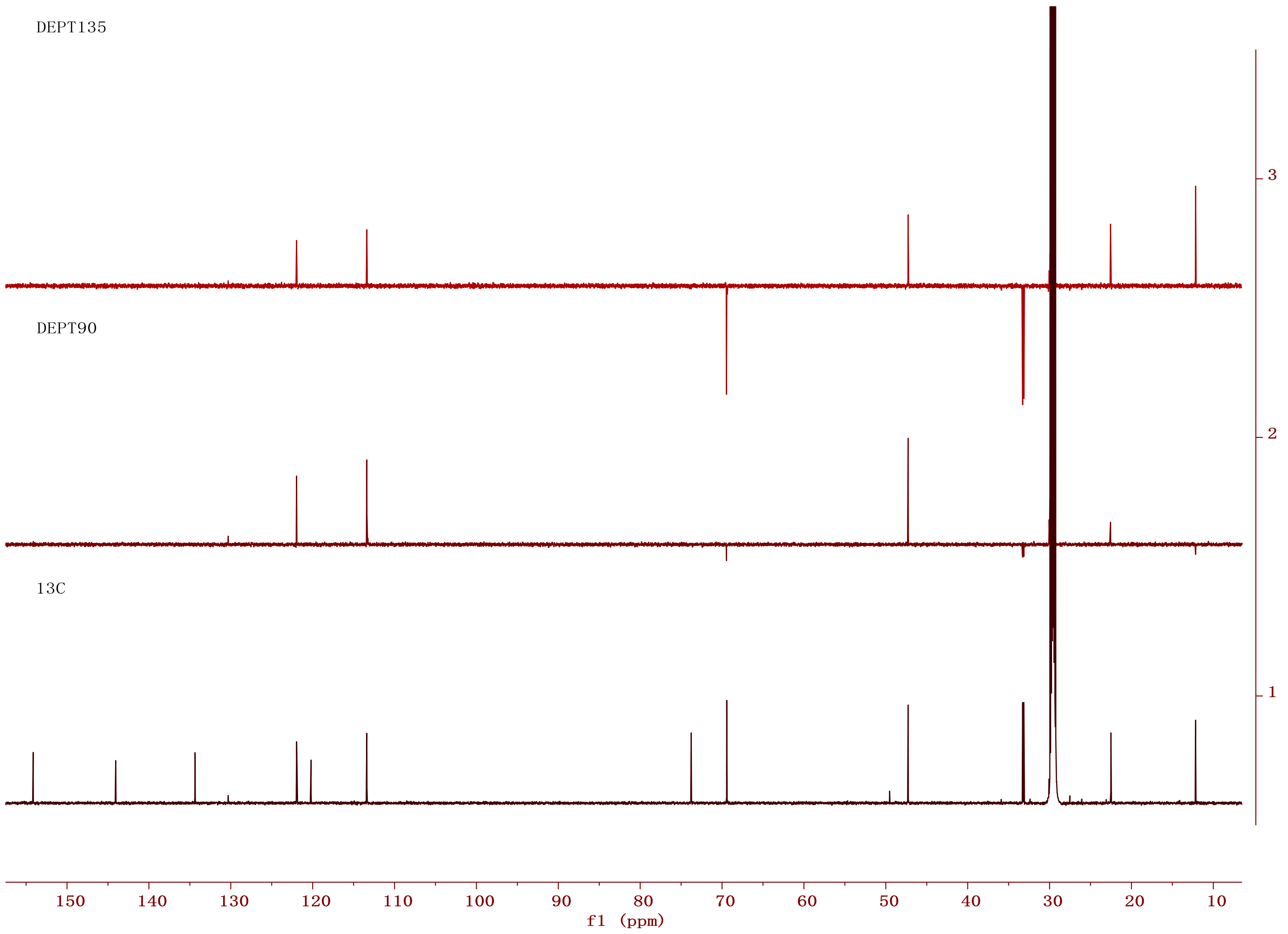

Figure S8. The DEPT Spectrum of Compound **1** in Acetone-d_6.

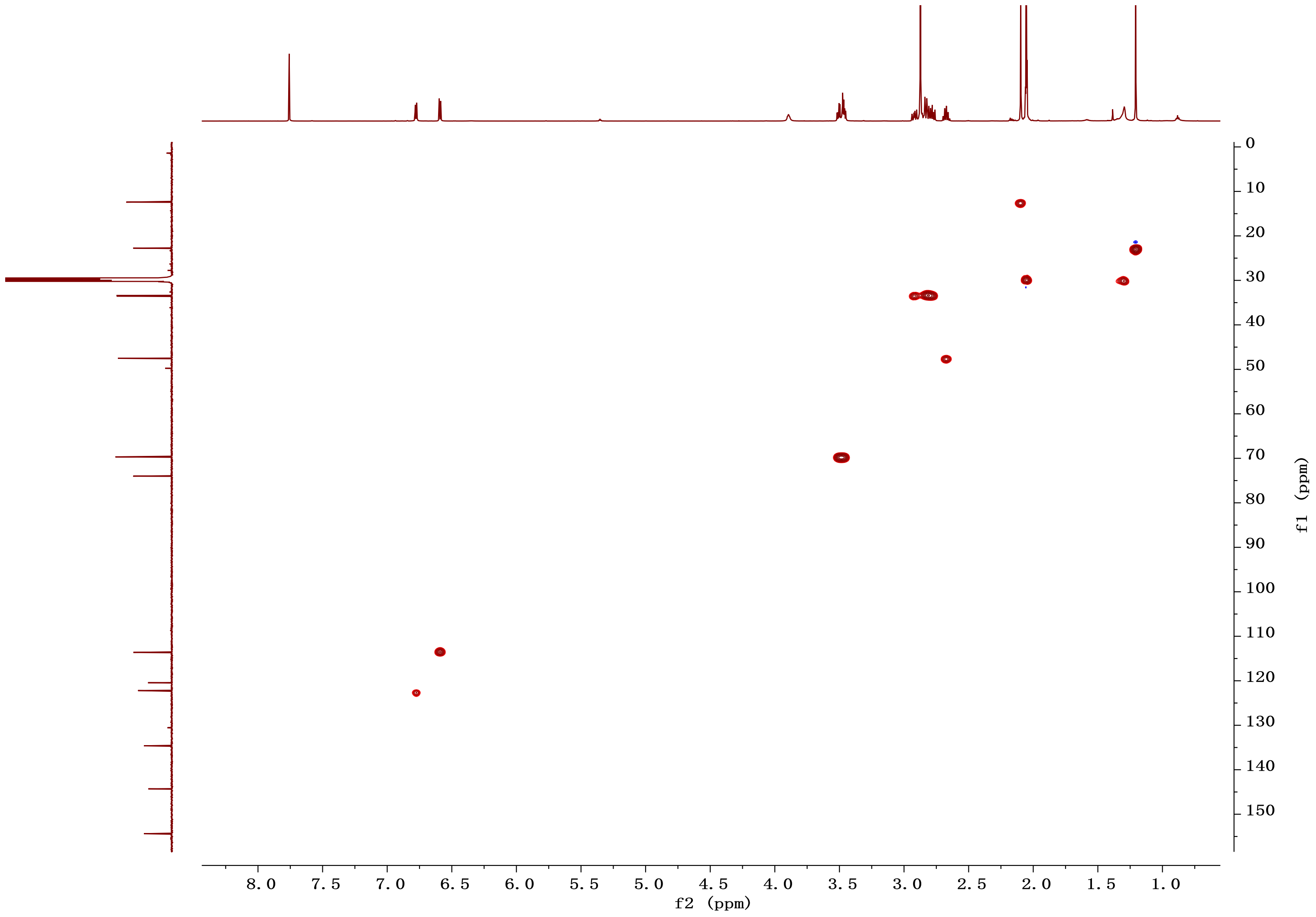

Figure S9. The HSQC Spectrum of Compound **1** in Acetone-d_6.

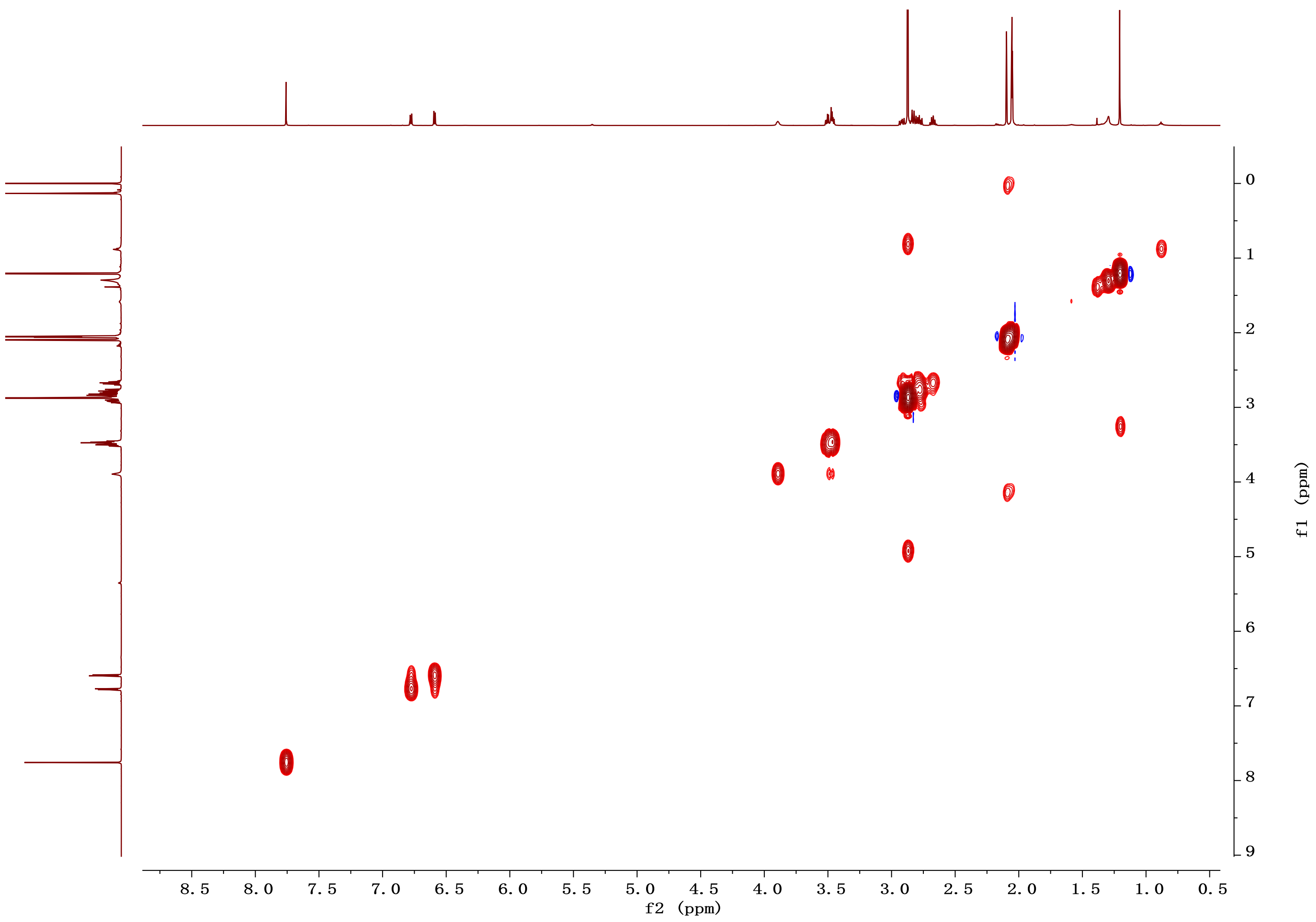

Figure S10. The ^{1}H-^{1}H COSY Spectrum of Compound **1** in Acetone-d_6.

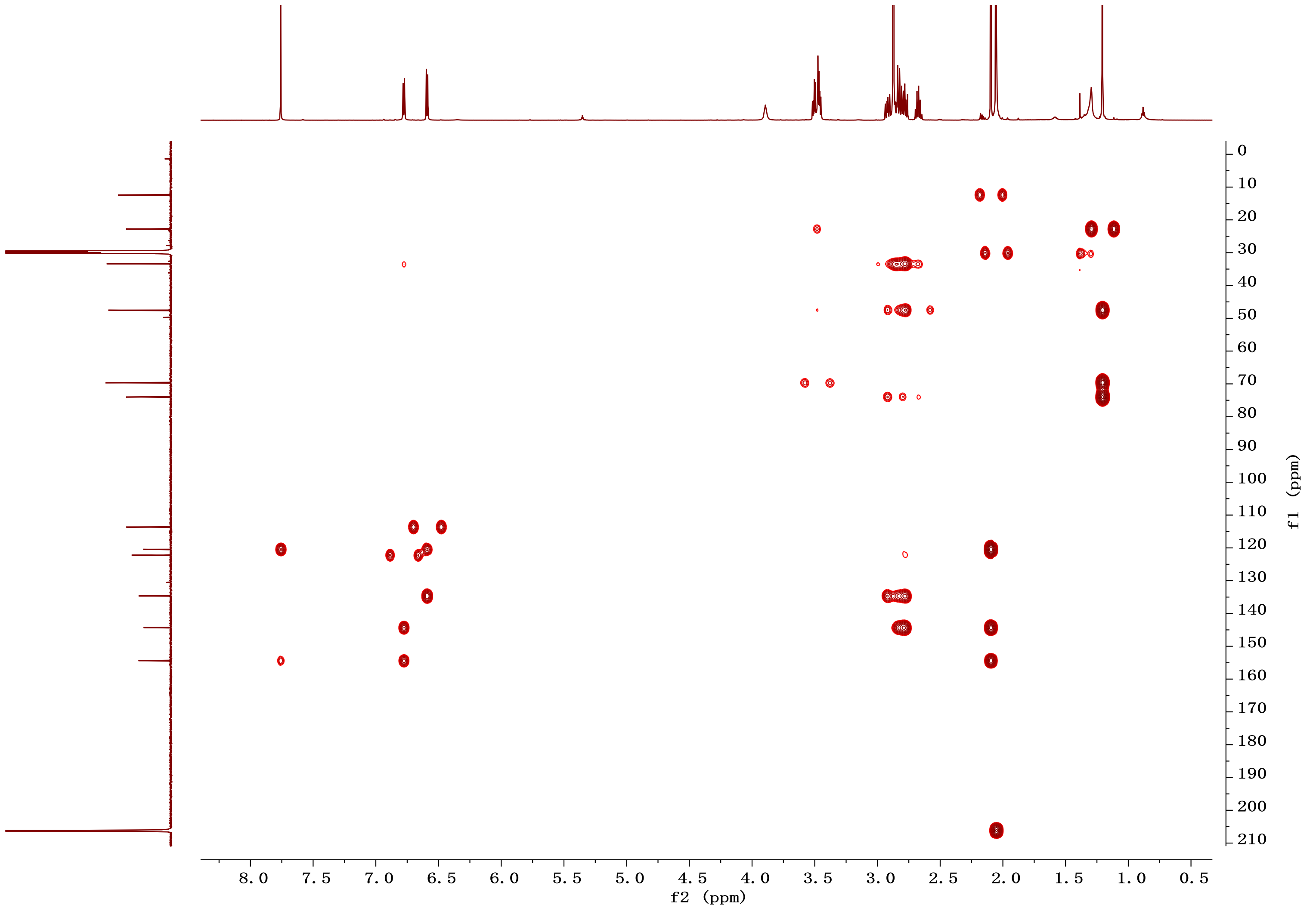

Figure S11. The HMBC Spectrum of Compound **1** in Acetone-d_6.

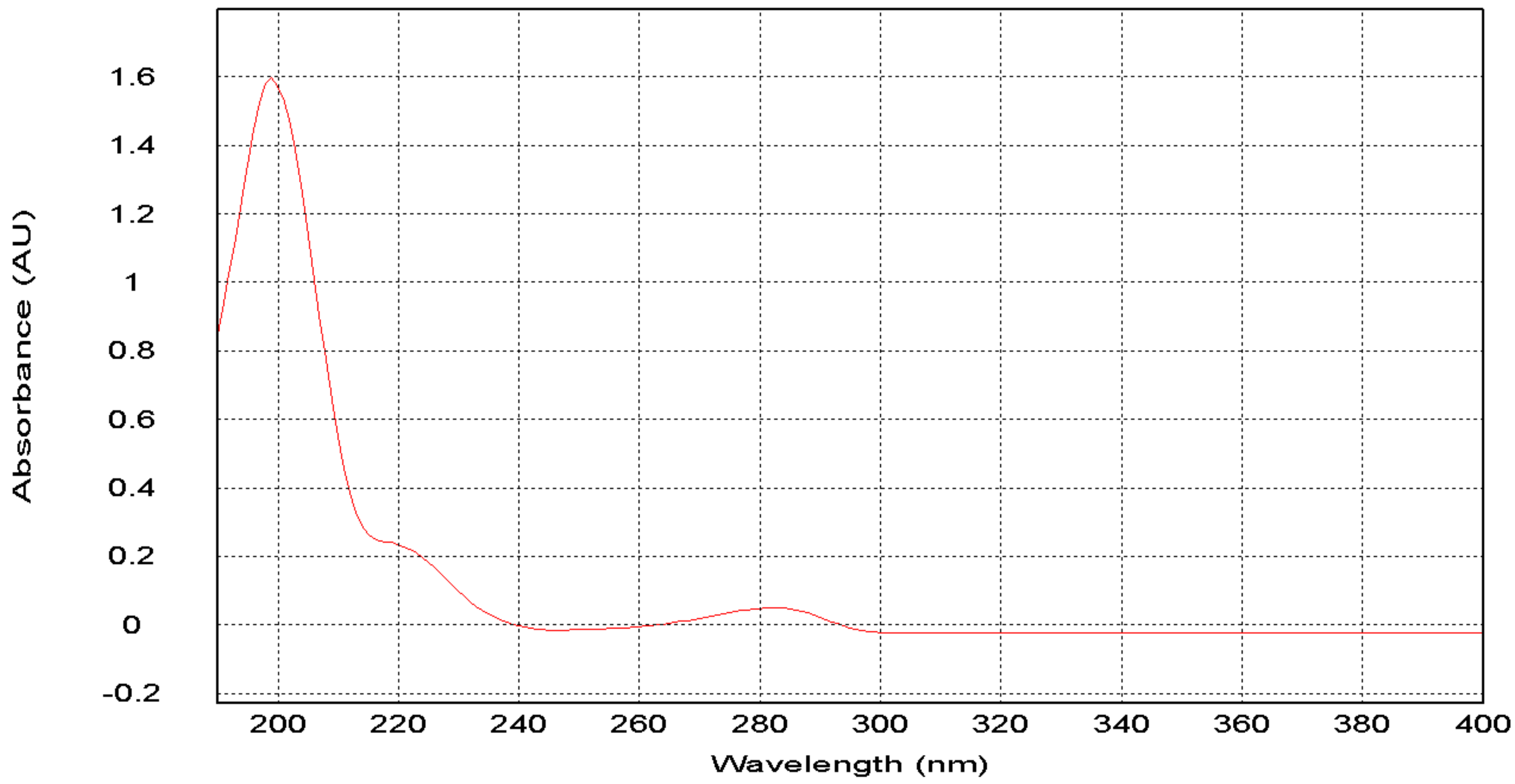

Figure S12. The UV spectrum of compound **2** in MeCN.

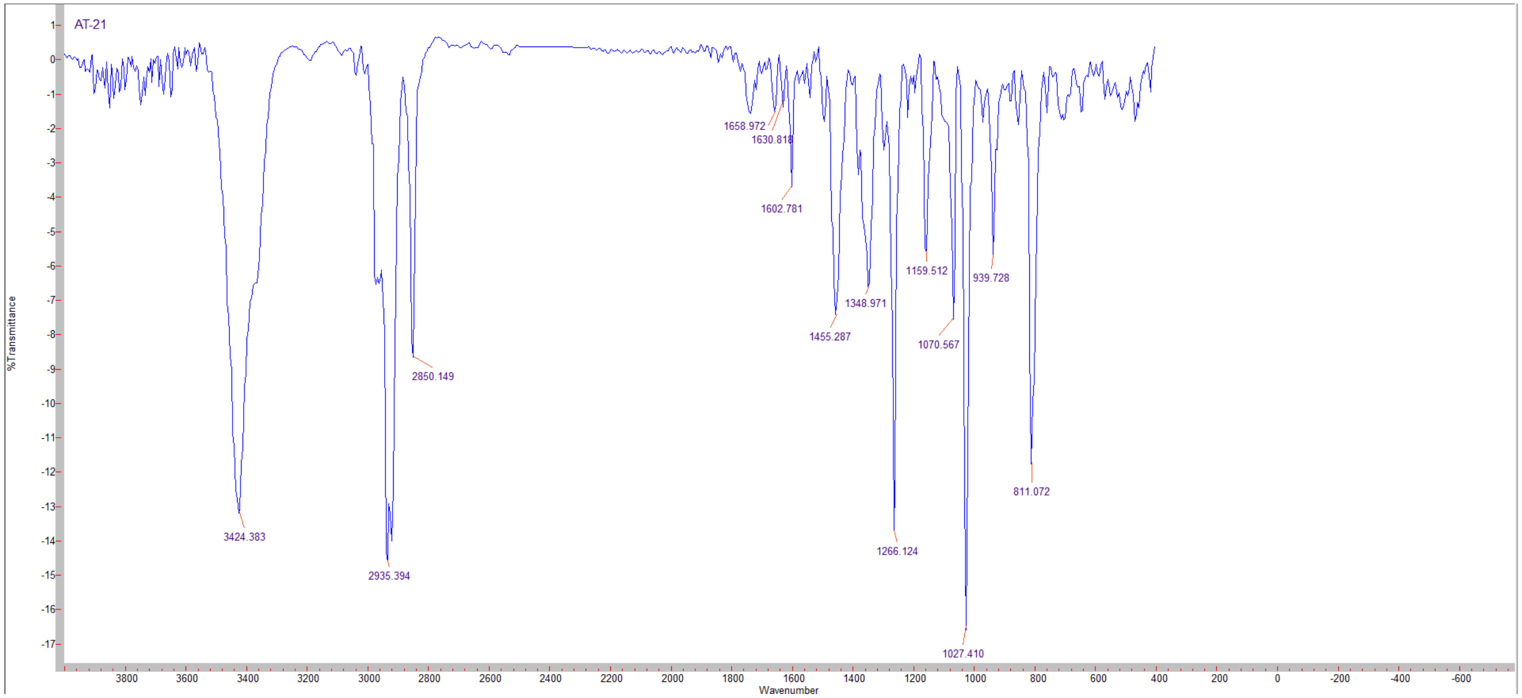

Figure S13. The IR Spectrum of Compound **2**.

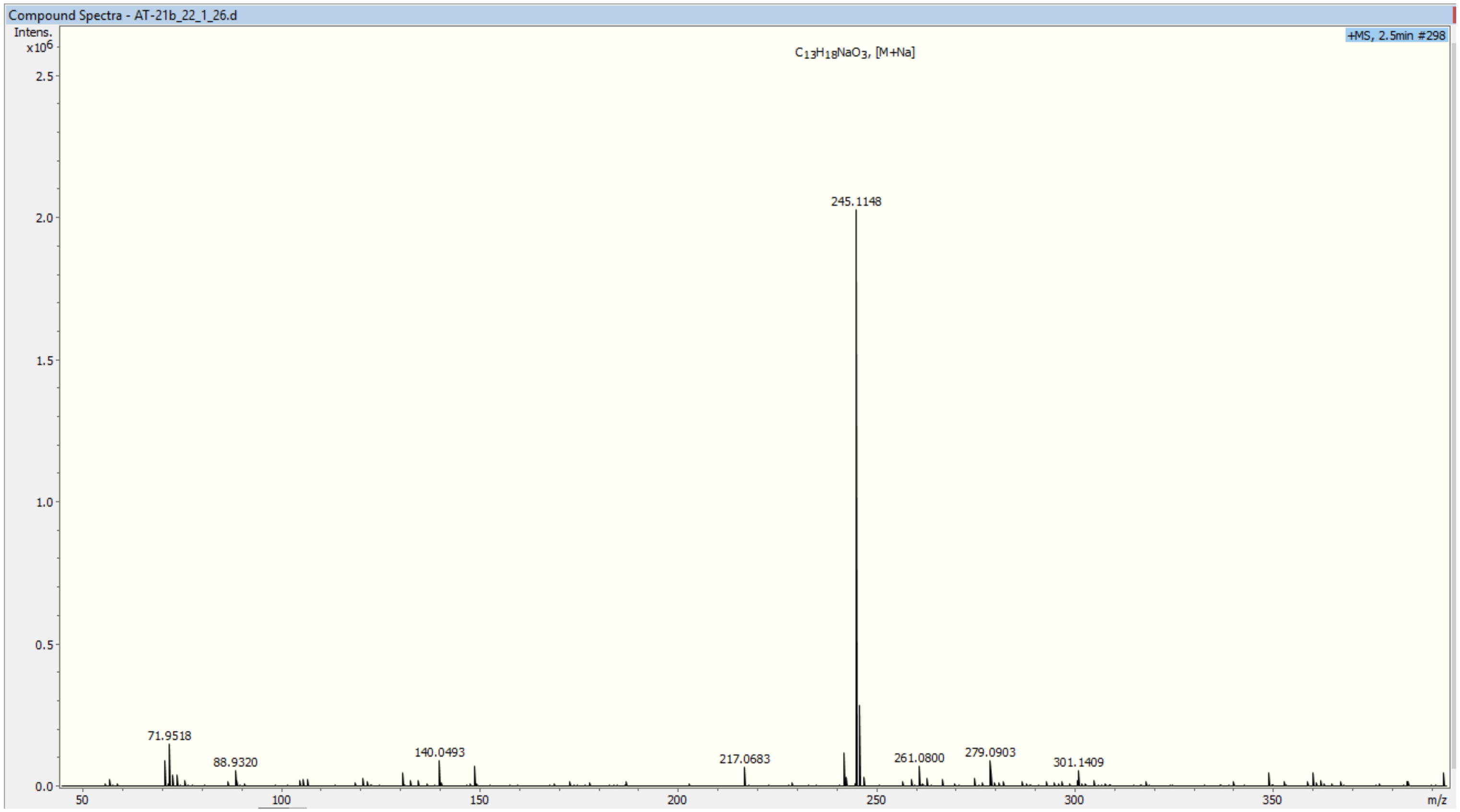

Figure S14. The (+)-HR-ESI-MS Spectroscopic Data of Compound **2**.

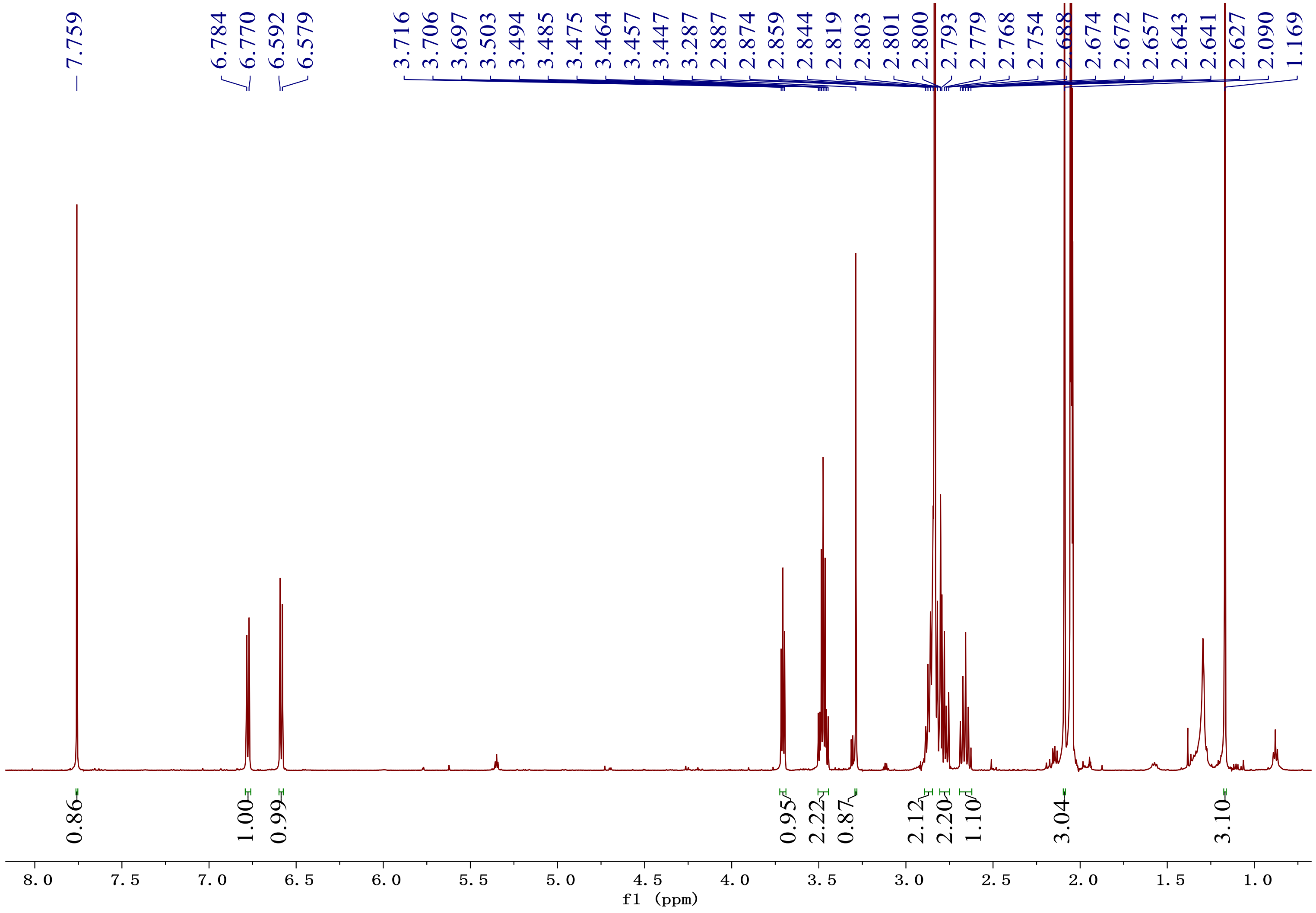

Figure S15. The ^{1}H NMR Spectrum of Compound **2** in Acetone-d_6.

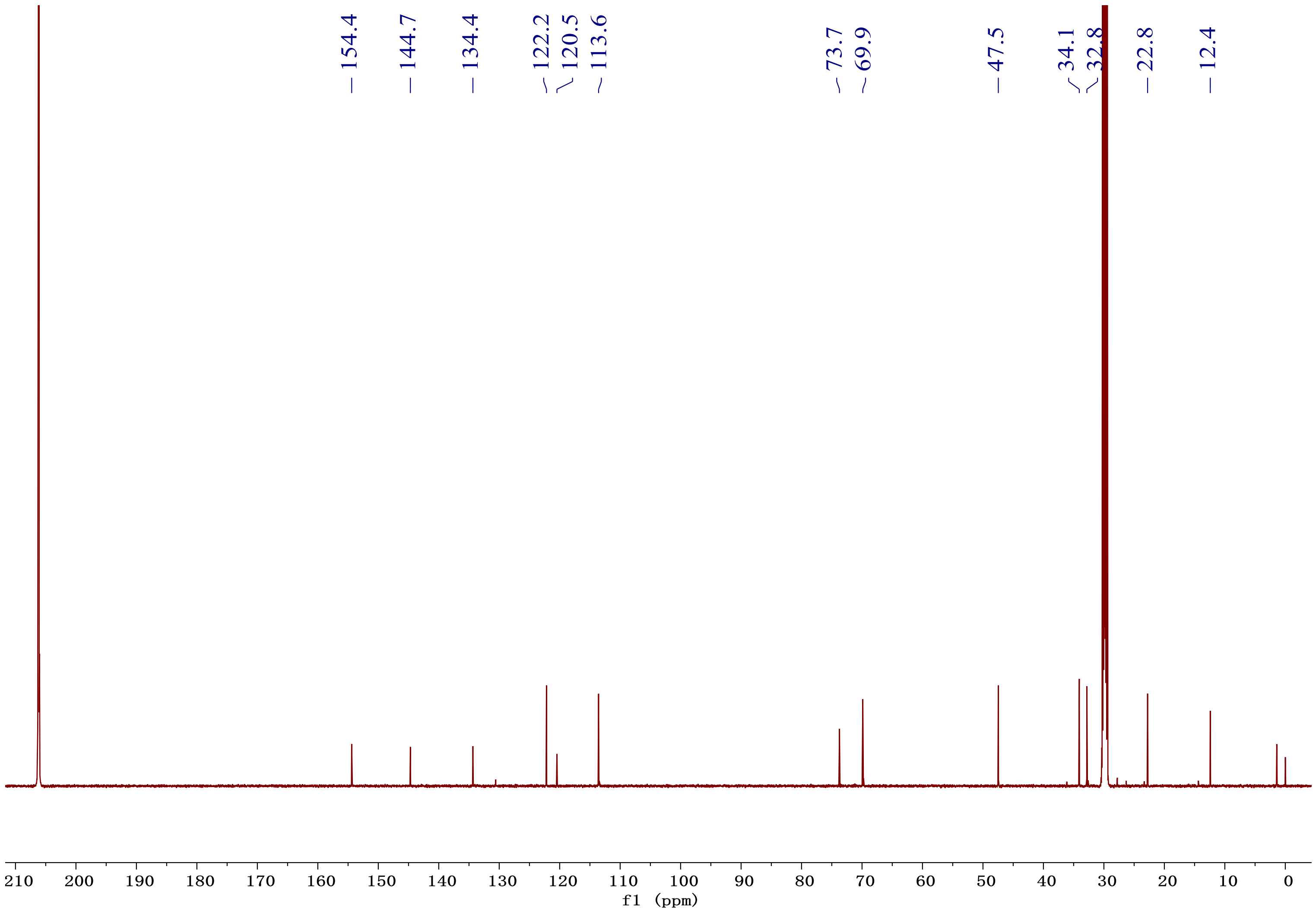

Figure S16. The ^{13}C NMR Spectrum of Compound **2** in Acetone-d_6.

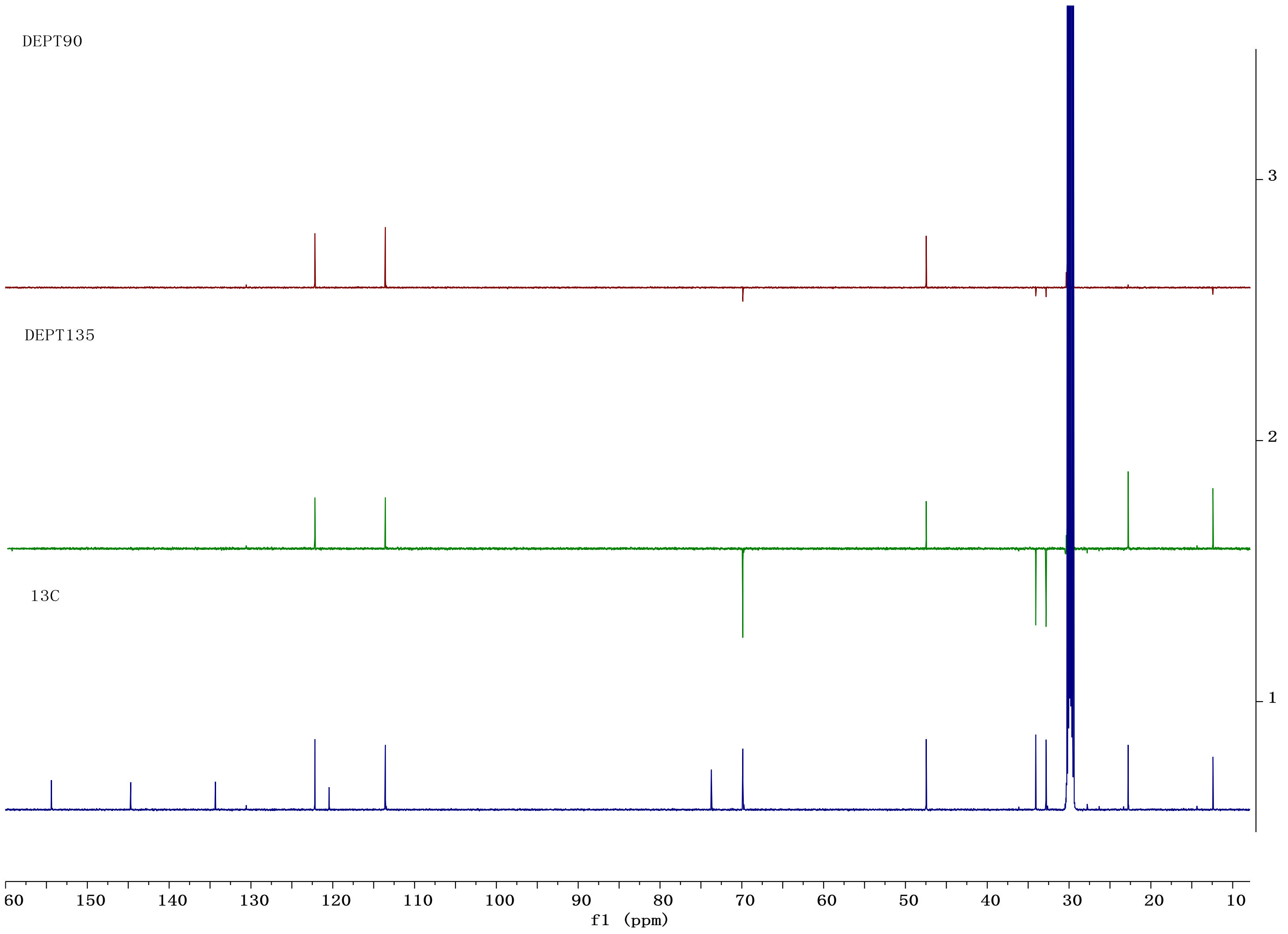

Figure S17. The DEPT Spectrum of Compound **2** in Acetone-d_6.

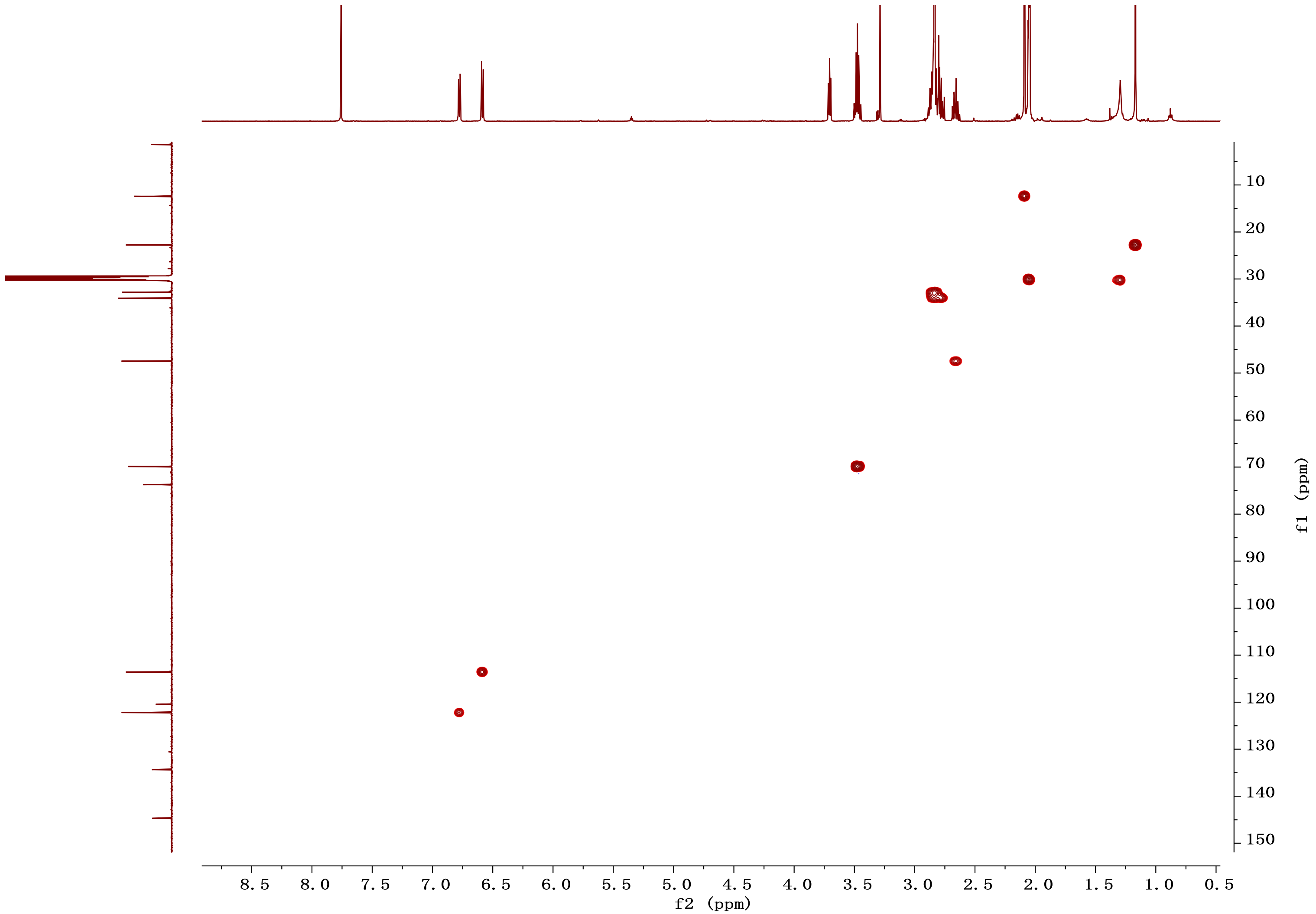

Figure S18. The HSQC Spectrum of Compound **2** in Acetone-d_6.

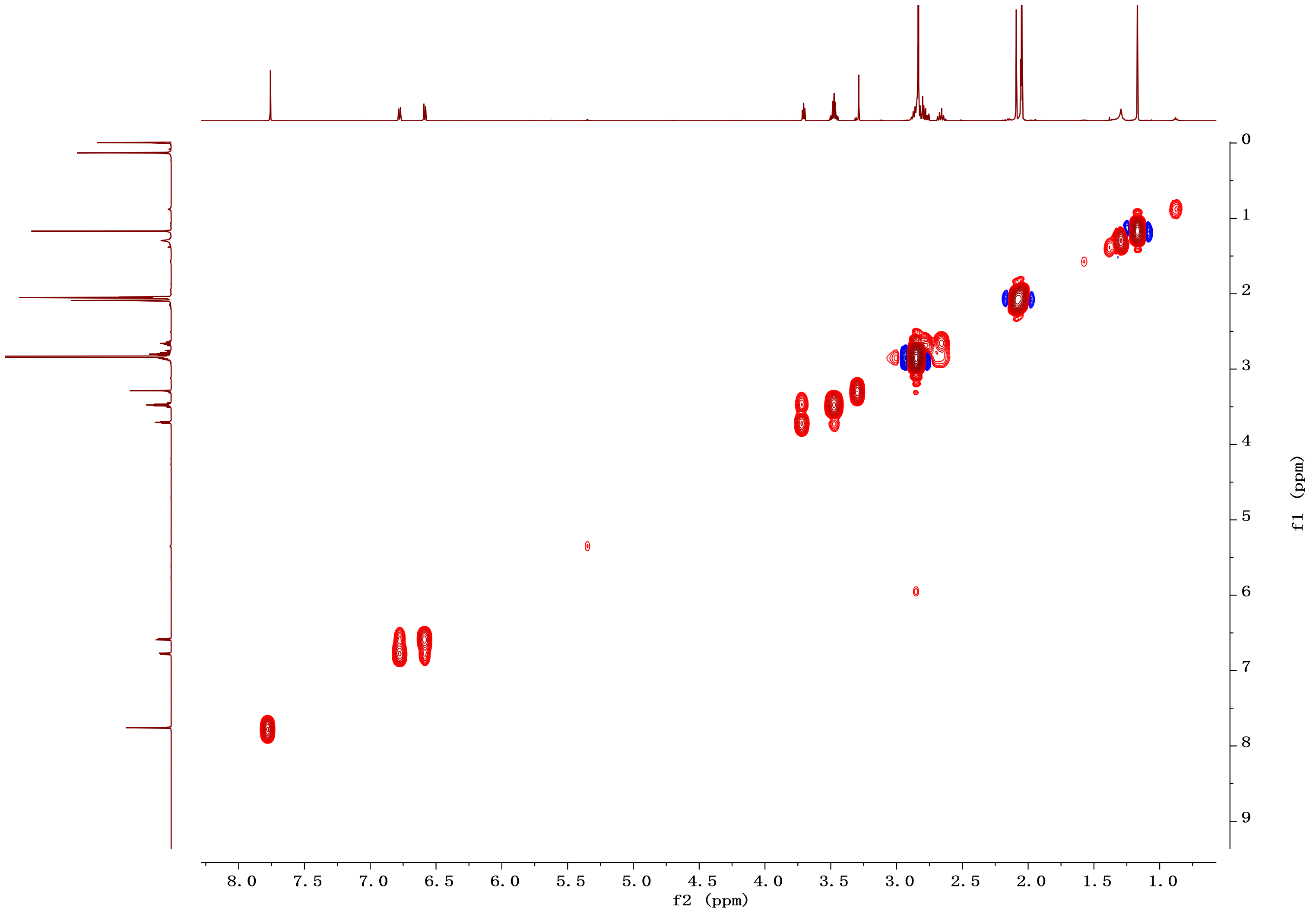

Figure S19. The ^{1}H-^{1}H COSY Spectrum of Compound **2** in Acetone-d_6.

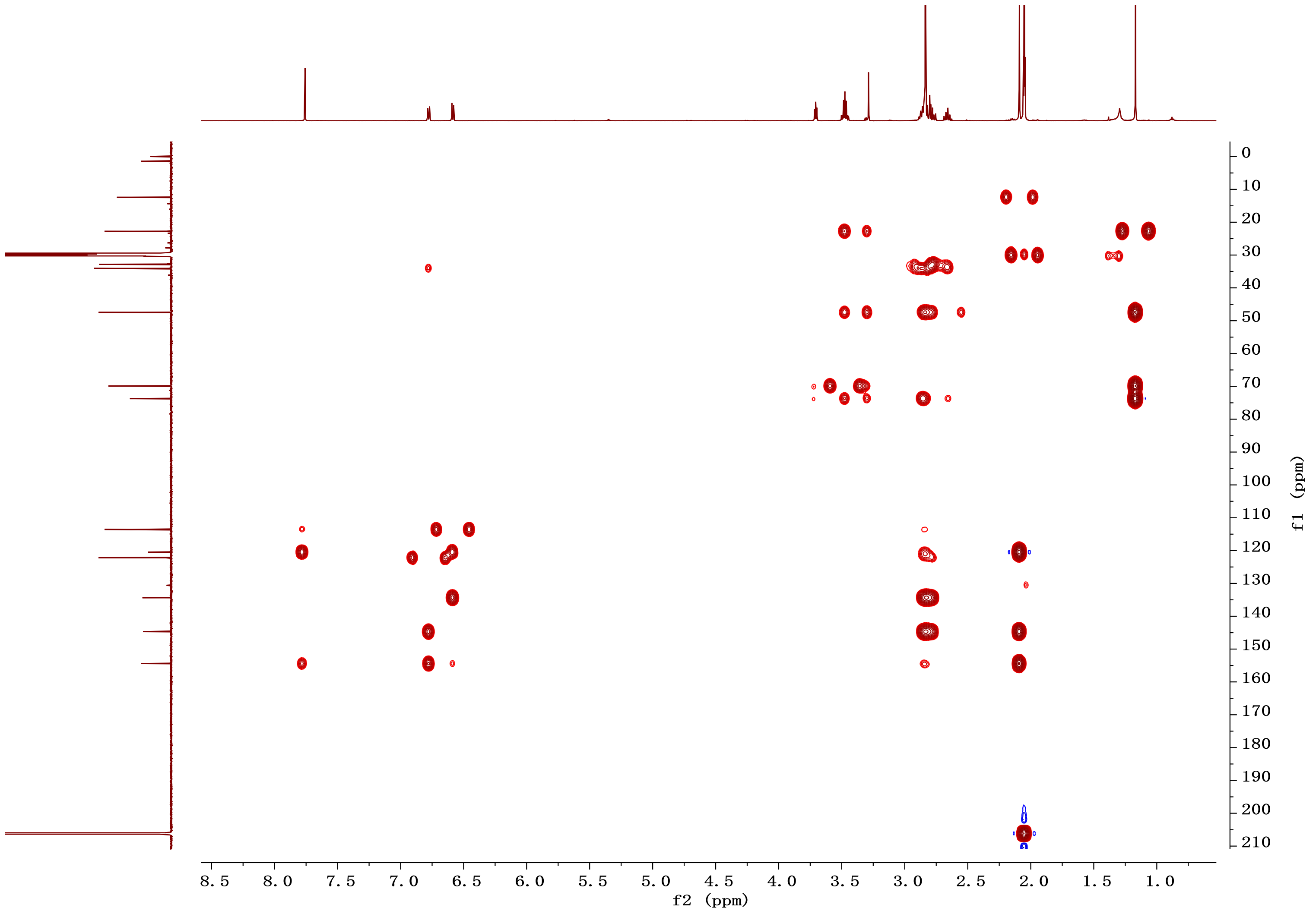

Figure S20. The HMBC Spectrum of Compound **2** in Acetone-d_6.

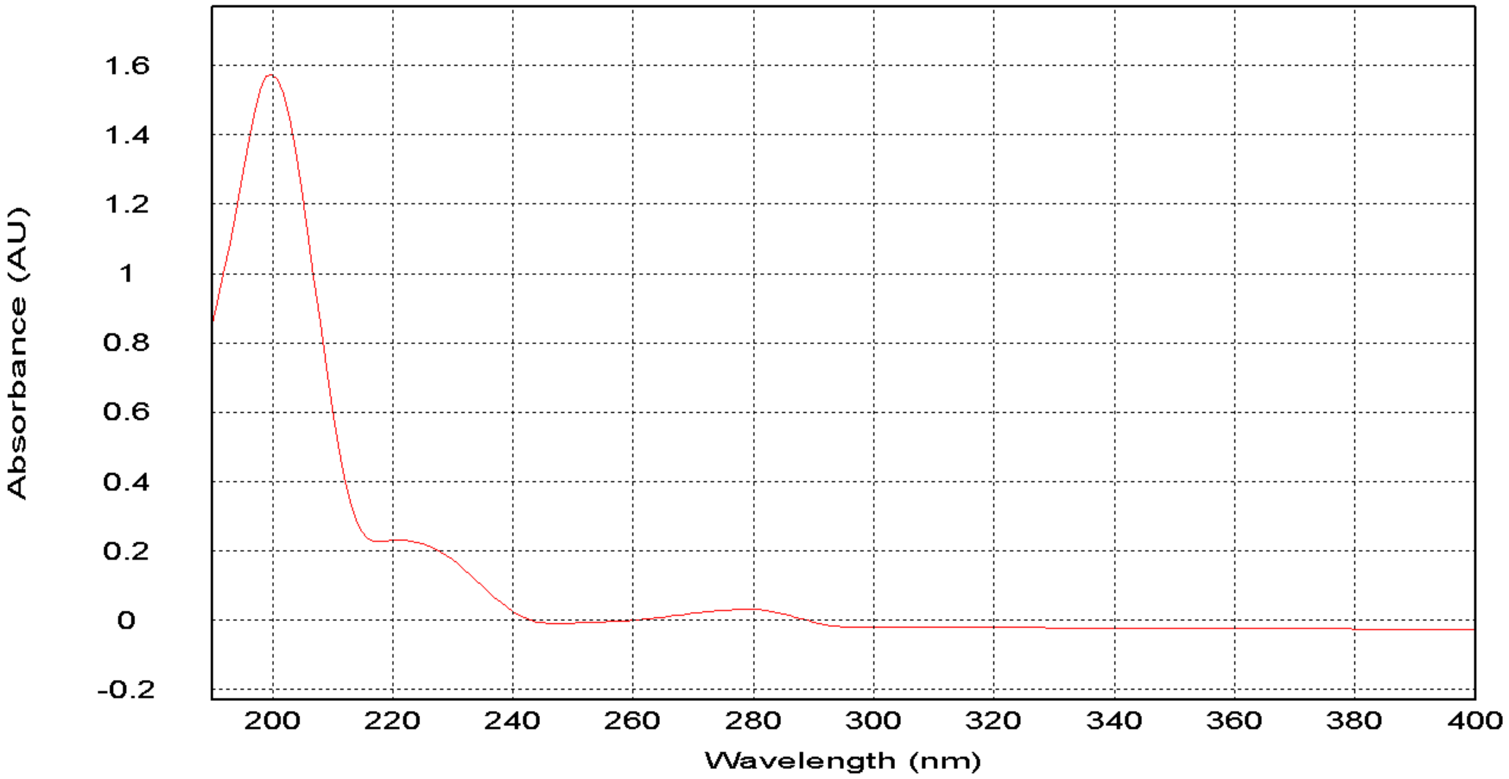

Figure S21. The UV spectrum of compound **3** in MeCN.

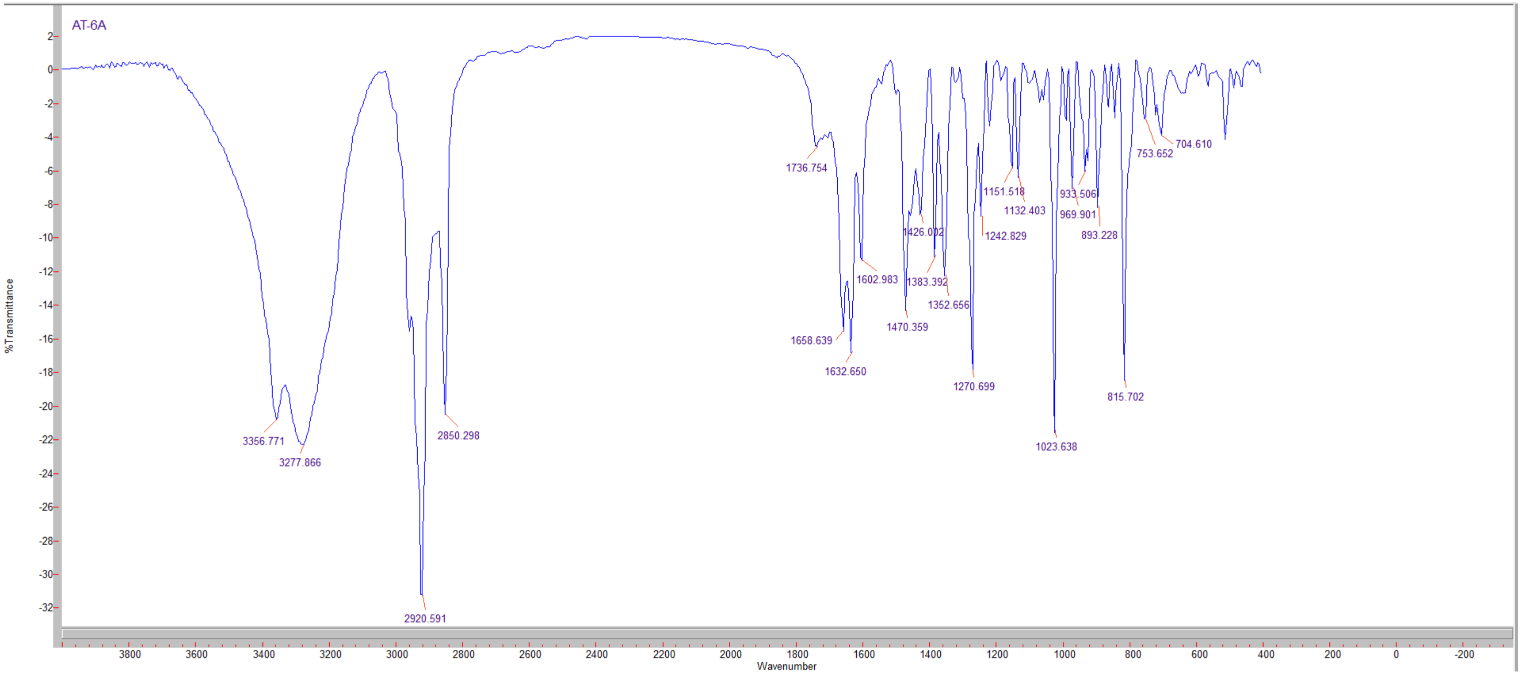

Figure S22. The IR Spectrum of Compound **3**.

Single Mass Analysis
Tolerance = 0.6 mDa / DBE: min = -1.5, max = 50.0
Element prediction: Off
Number of isotope peaks used for i-FIT = 3
Monoisotopic Mass, Even Electron Ions
74 formula(e) evaluated with 1 results within limits (up to 50 best isotopic matches for each mass)
Elements Used:

Mass	Calc. Mass	mDa	PPM	DBE	Formula	i-FIT	i-FIT Norm	Fit Conf %	C	H	O	Na
243.0996	243.0997	-0.1	-0.4	5.5	C13 H16 O3 Na	103.9	n/a	n/a	13	16	3	1

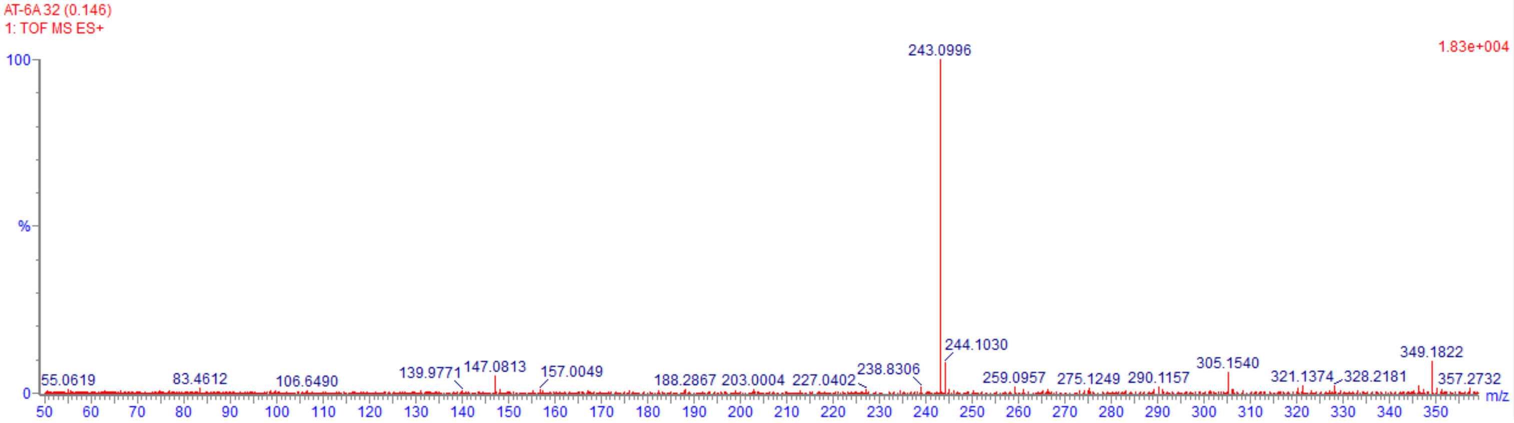

Figure S23. The (+)-HR-ESI-MS Spectroscopic Data of Compound **3**.

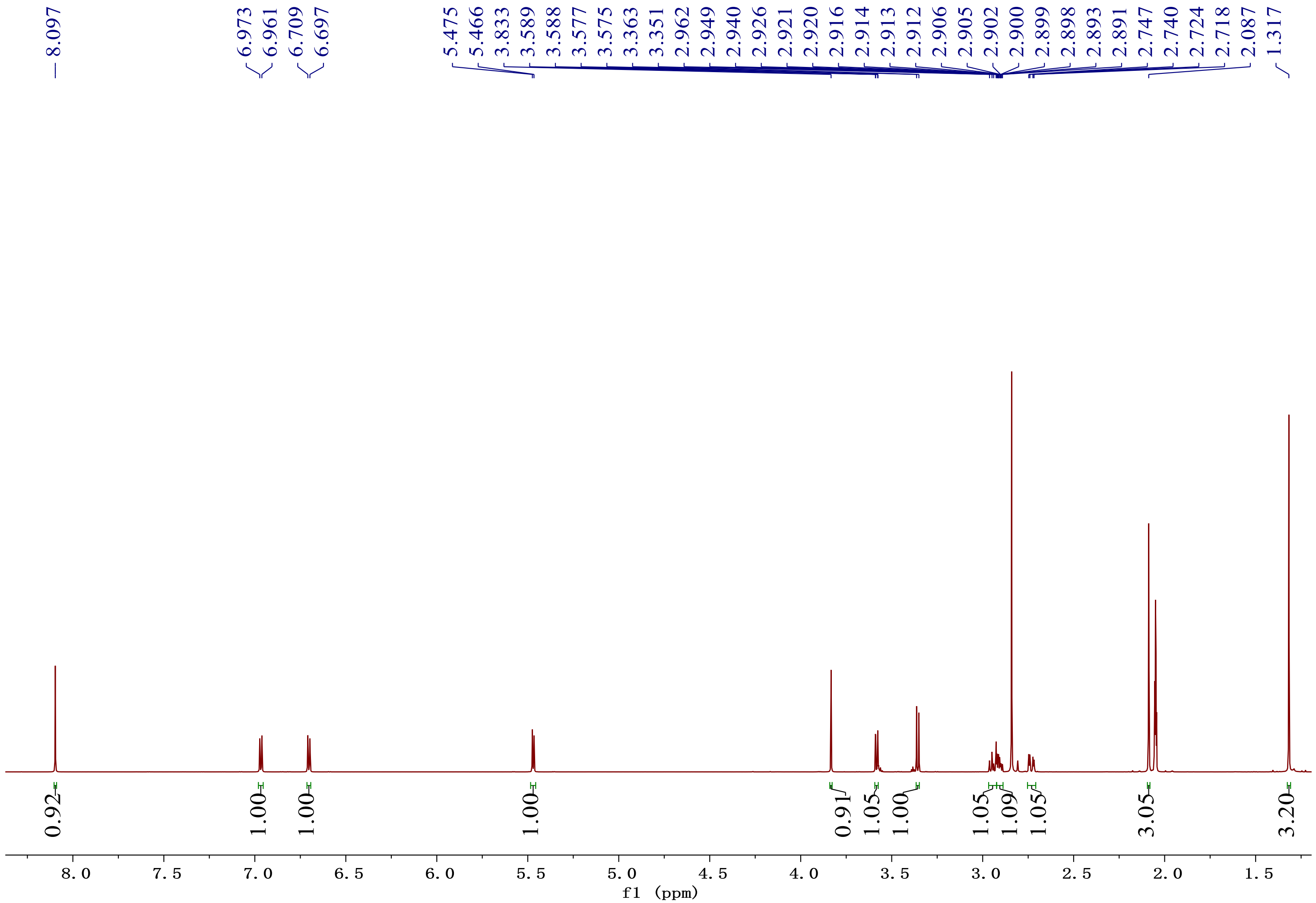

Figure S24. The ^{1}H NMR Spectrum of Compound **3** in Acetone-d_6.

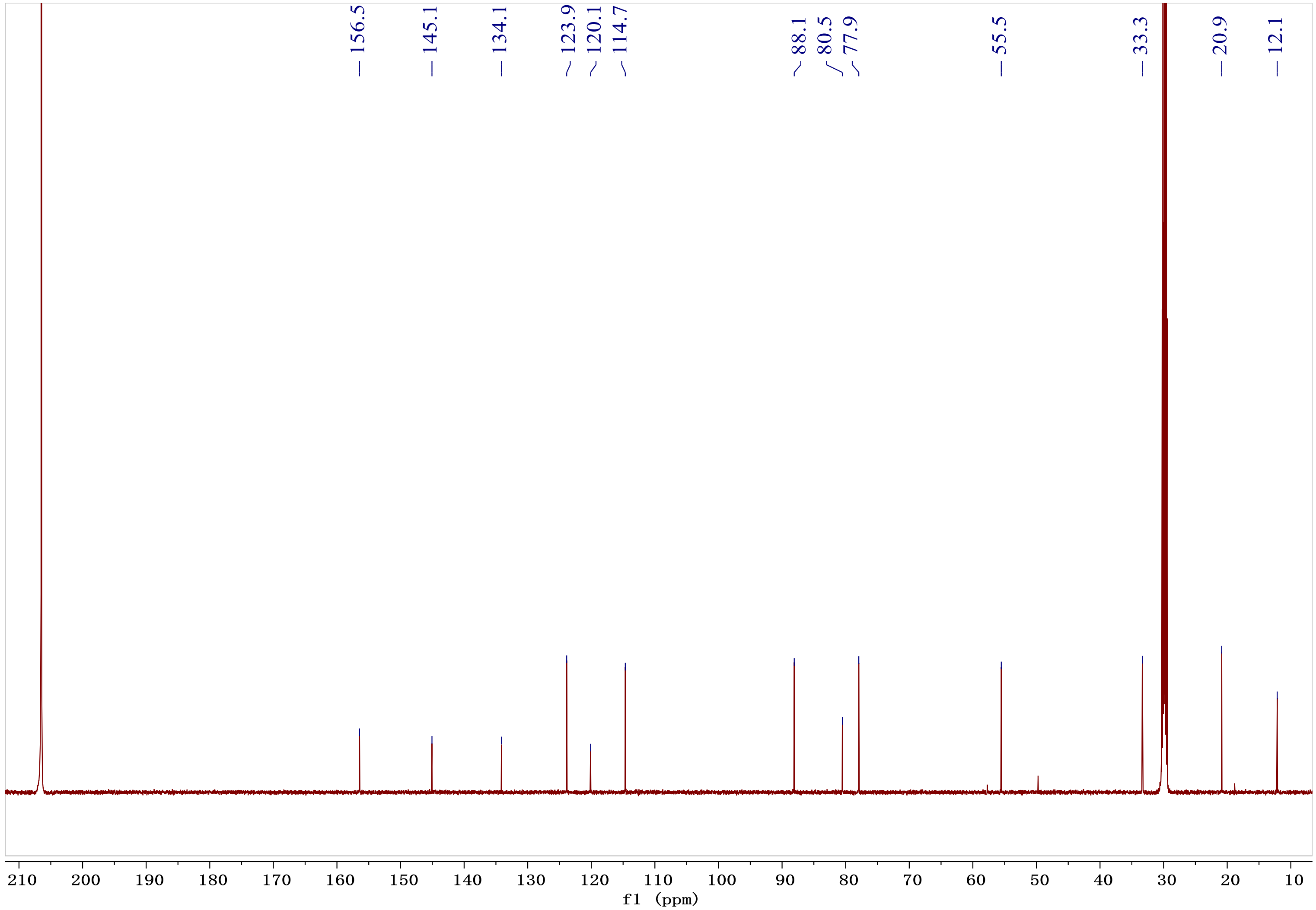

Figure S25. The [1]C NMR Spectrum of Compound **3** in Acetone-d_6.

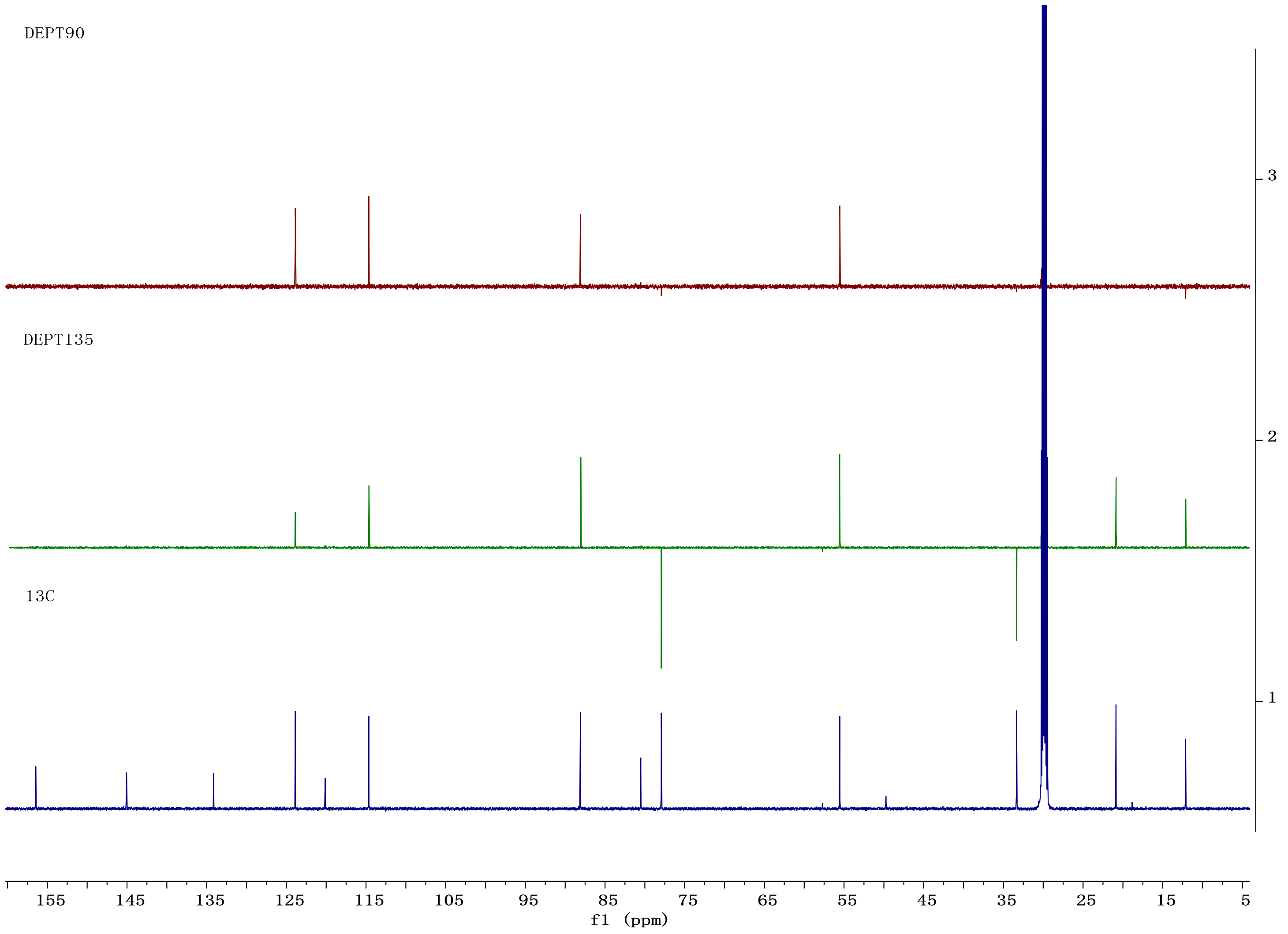

Figure S26. The DEPT Spectrum of Compound **3** in Acetone-d_6.

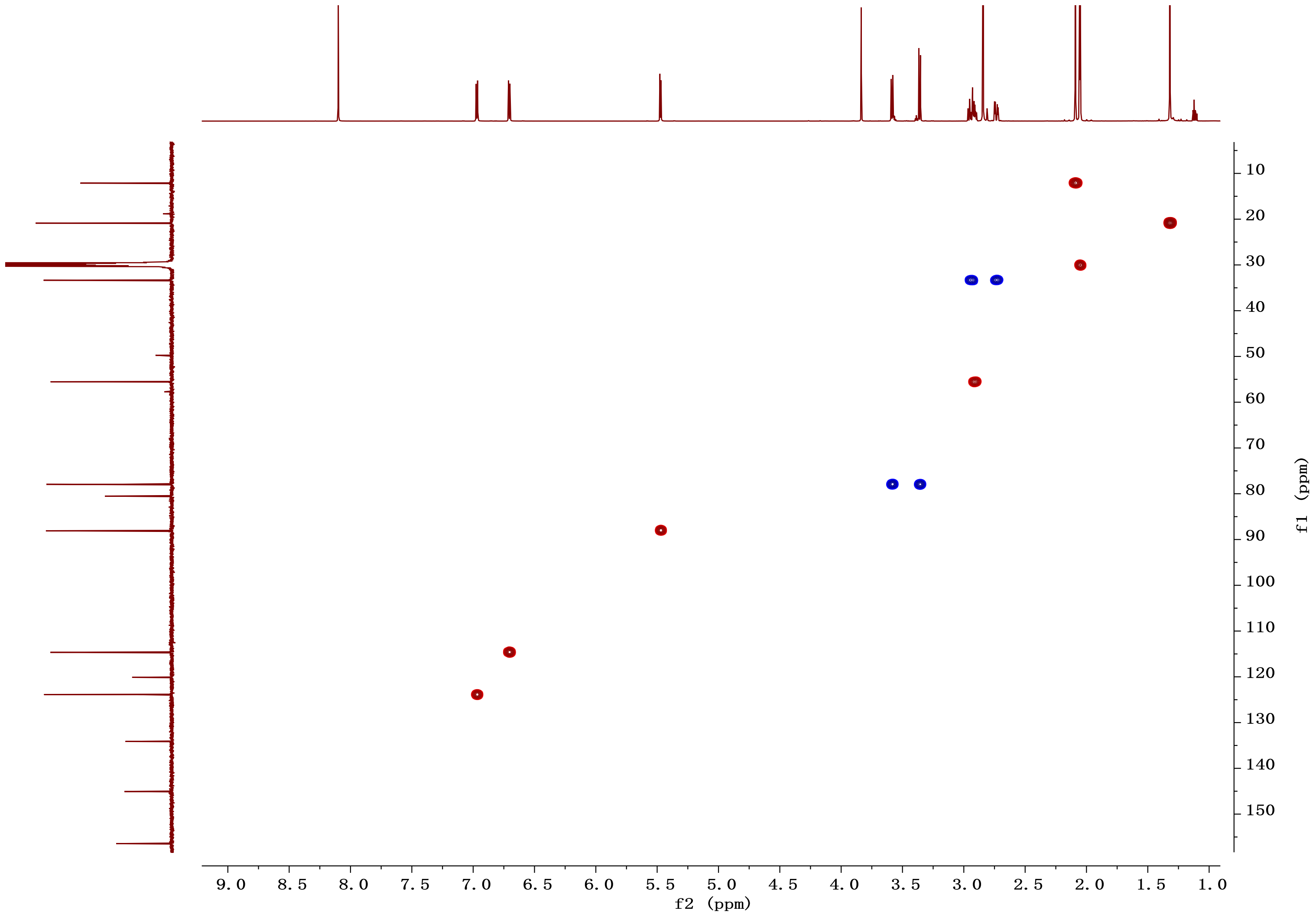

Figure S27. The edit-HSQC Spectrum of Compound **3** in Acetone-d_6.

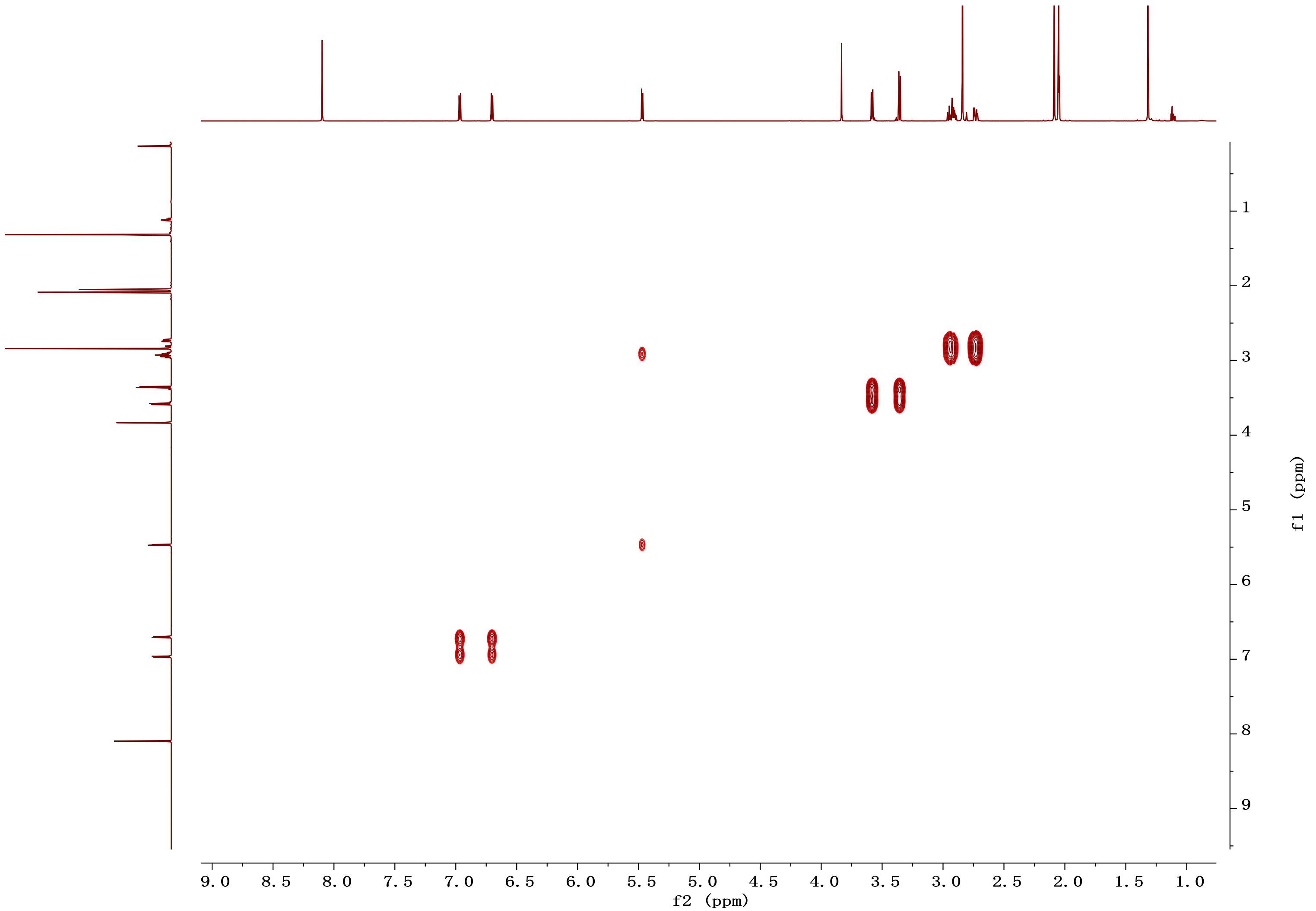

Figure S28. The ^{1}H-^{1}H COSY Spectrum of Compound **3** in Acetone-d_6.

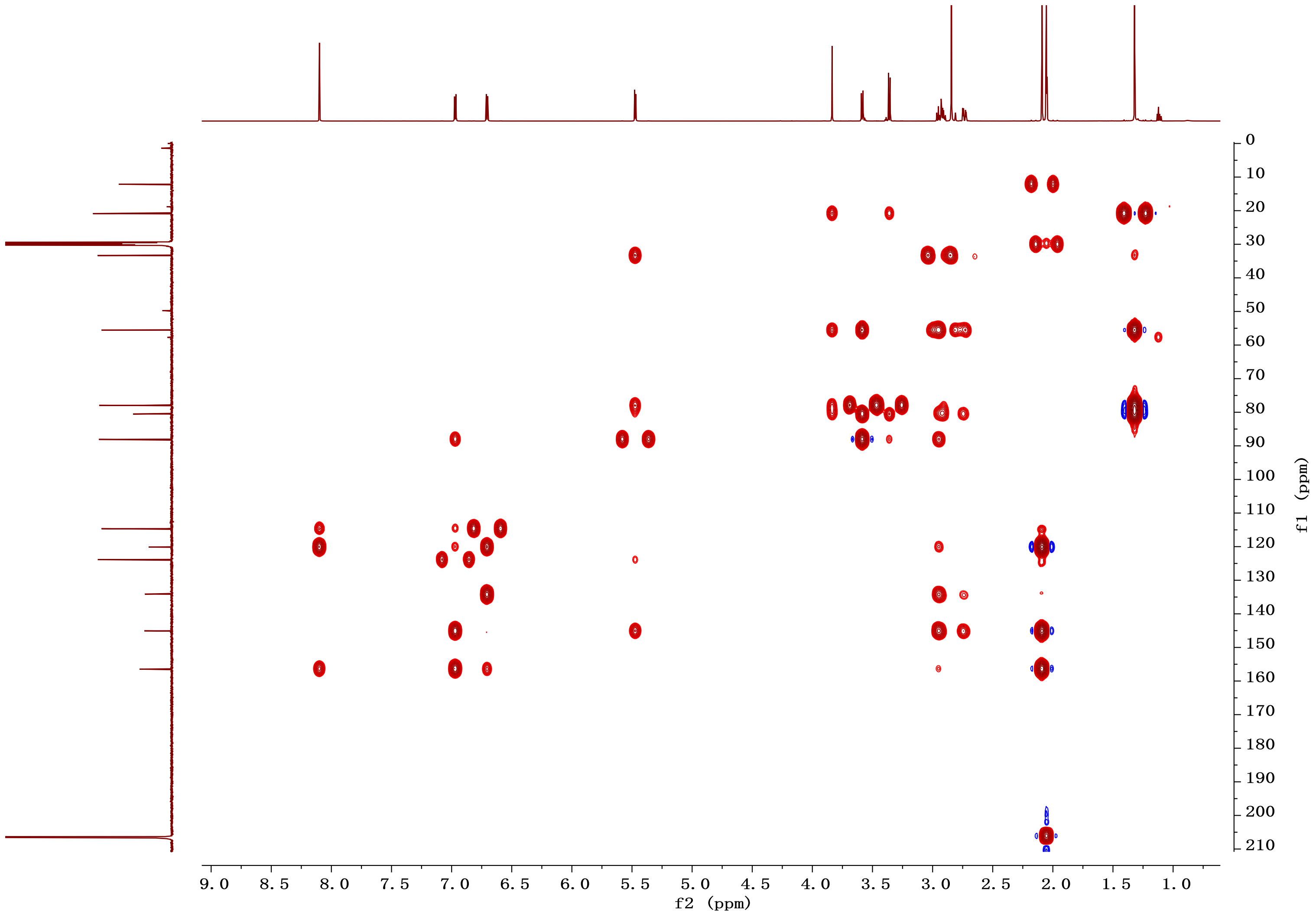

Figure S29. The HMBC Spectrum of Compound **3** in Acetone-d_6.

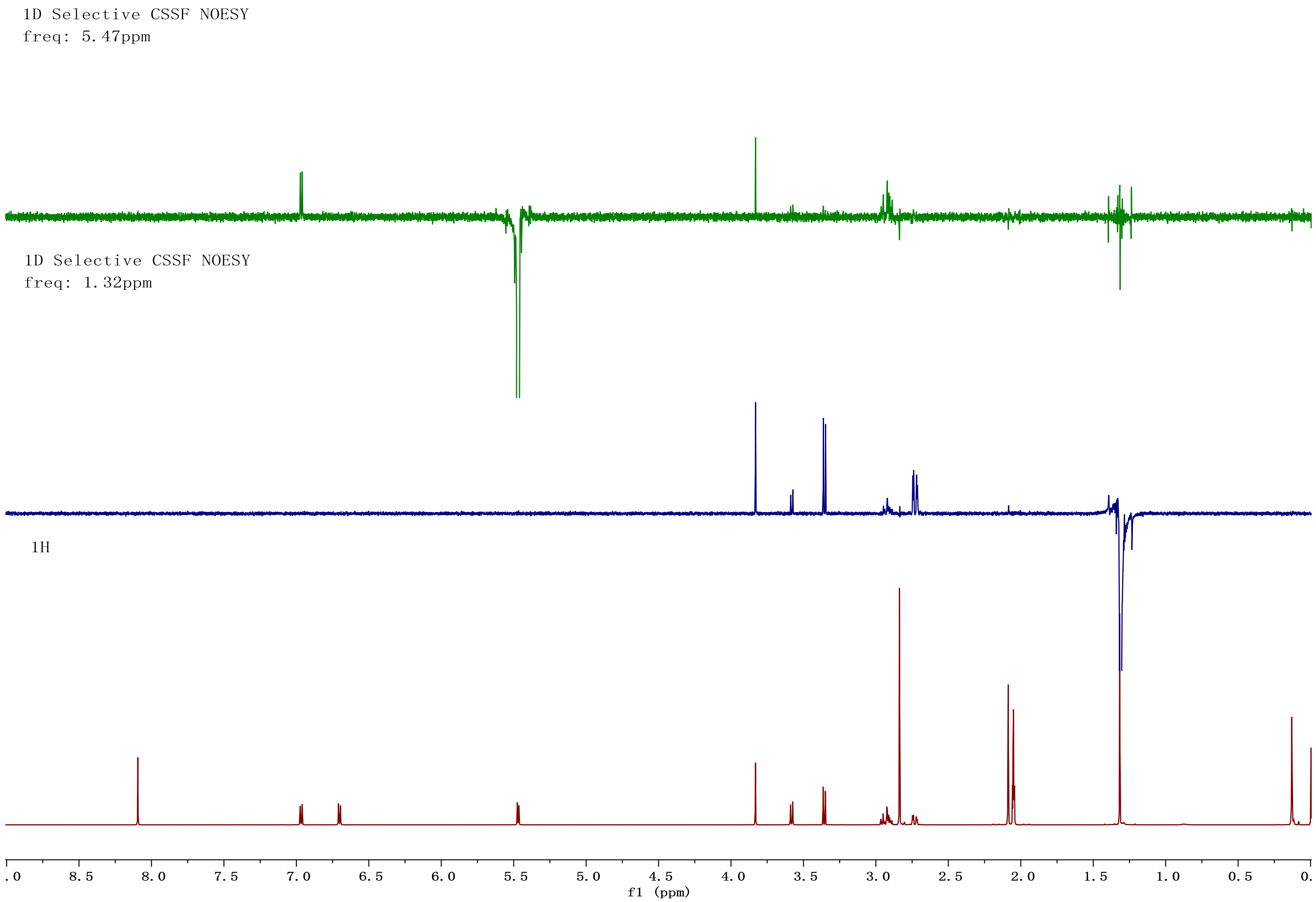

Figure S30. The 1D NOE Spectrum of Compound **3** in Acetone-d_6.

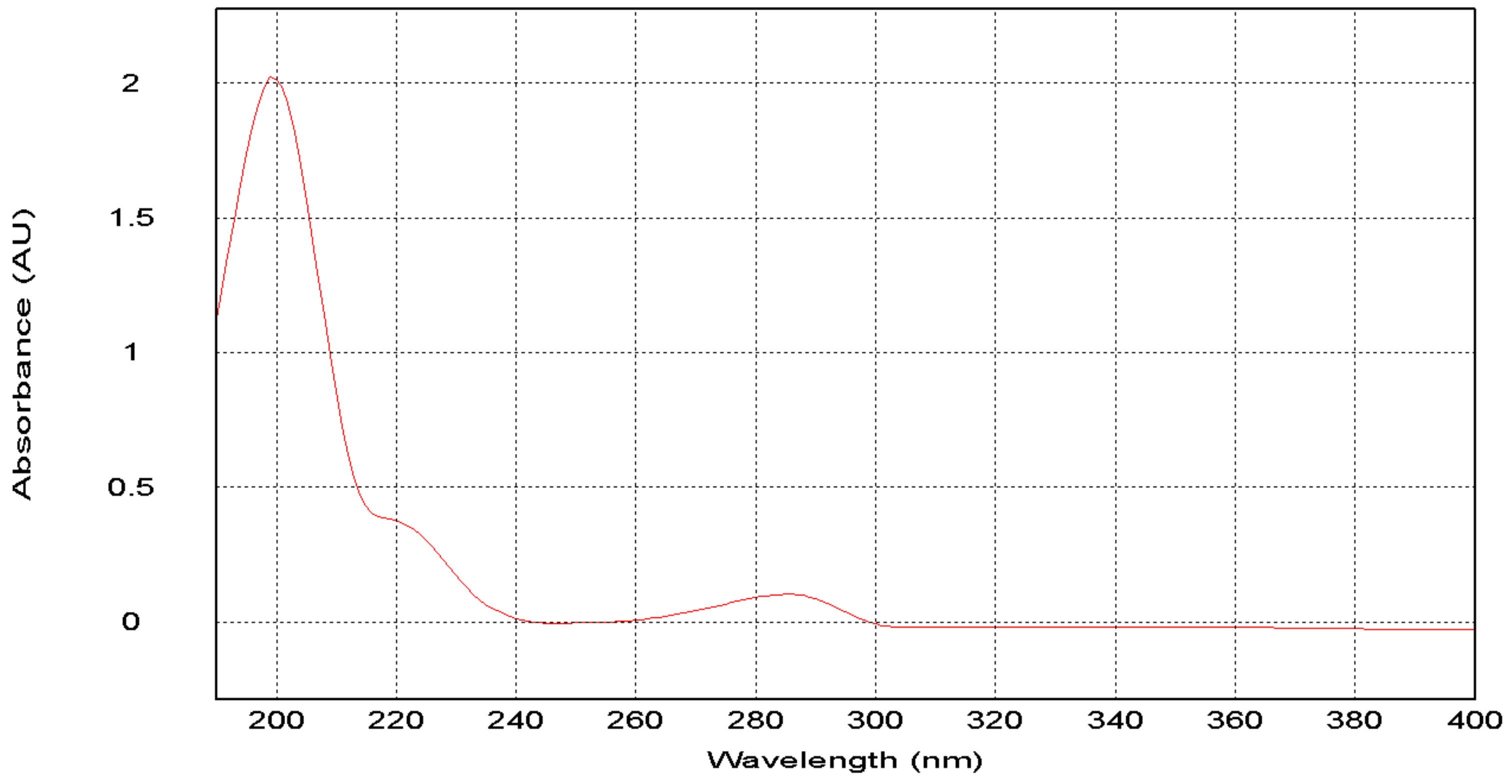

Figure S31. The UV spectrum of compound **4** in MeCN.

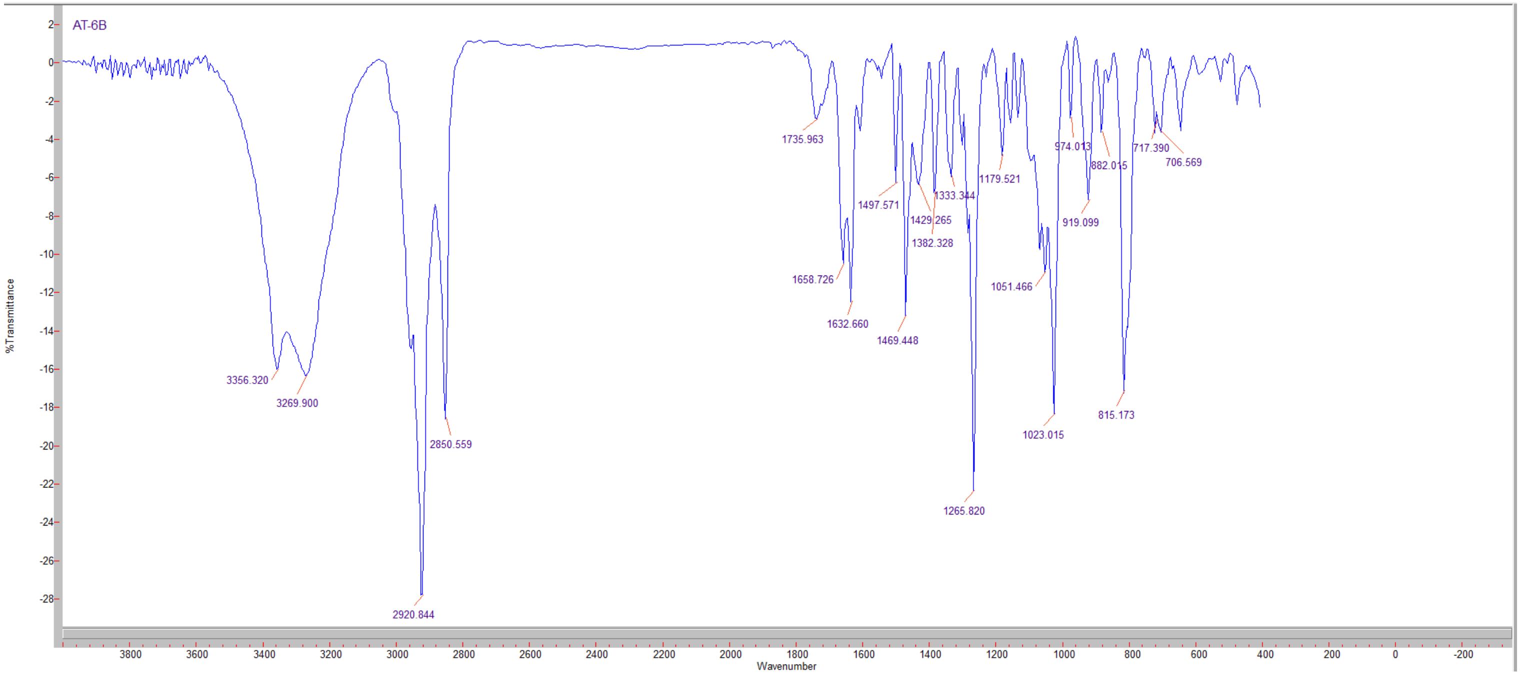

Figure S32. The IR Spectrum of Compound **4**.

Single Mass Analysis
Tolerance = 0.6 mDa / DBE: min = -1.5, max = 50.0
Element prediction: Off
Number of isotope peaks used for i-FIT = 3
Monoisotopic Mass, Even Electron Ions
74 formula(e) evaluated with 1 results within limits (up to 50 best isotopic matches for each mass)
Elements Used:

Mass	Calc. Mass	mDa	PPM	DBE	Formula	i-FIT	i-FIT Norm	Fit Conf %	C	H	O	Na
243.0997	243.0997	0.0	0.0	5.5	C13 H16 O3 Na	116.5	n/a	n/a	13	16	3	1

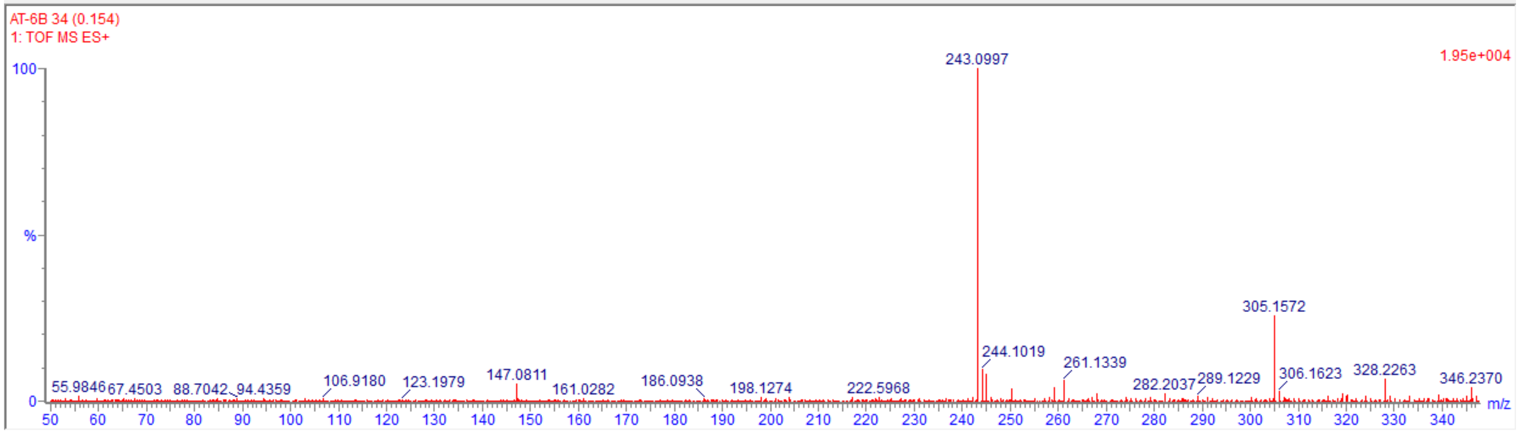

Figure S33. The (+)-HR-ESI-MS Spectroscopic Data of Compound **4**.

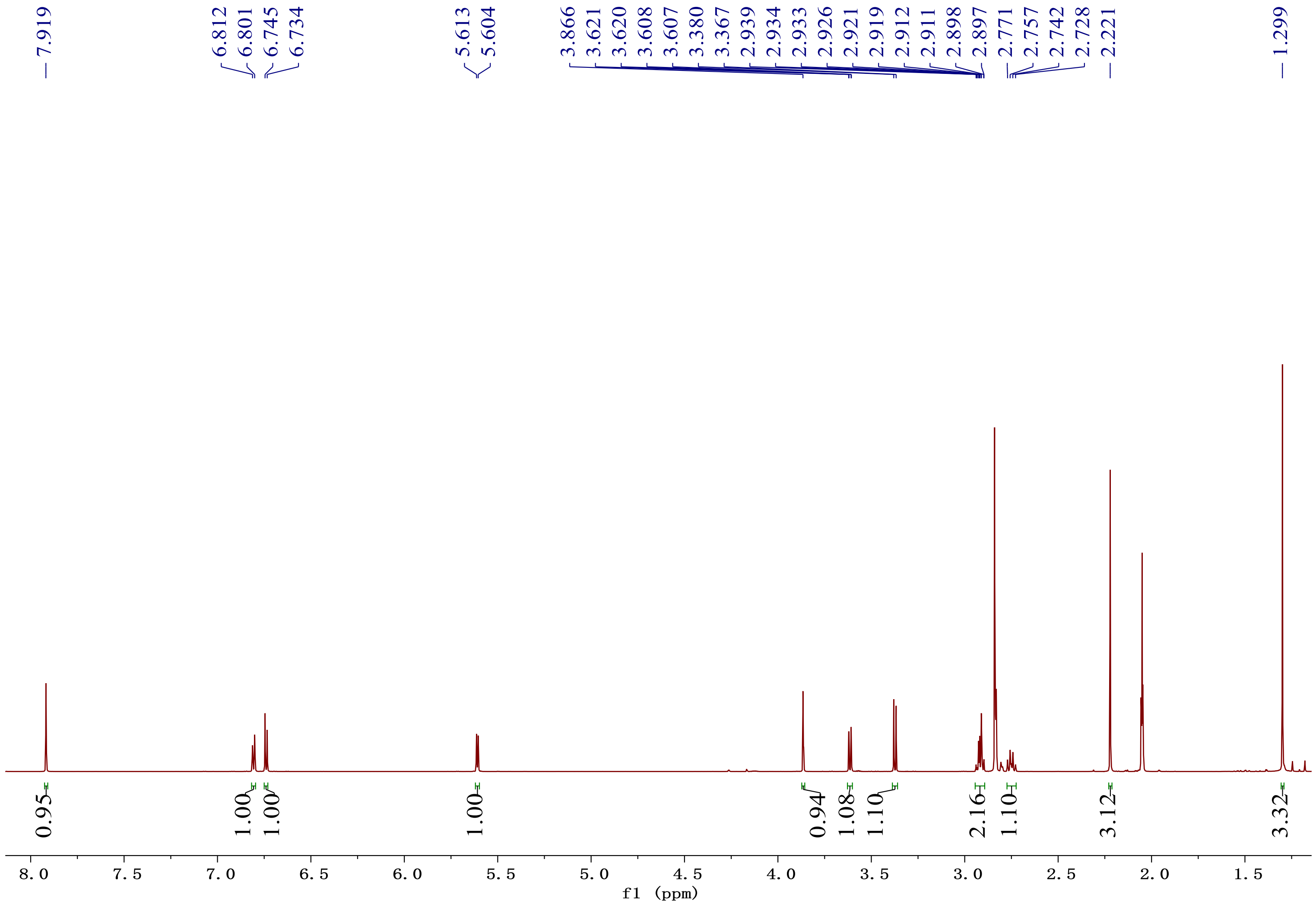

Figure S34. The ^{1}H NMR Spectrum of Compound **4** in Acetone-d_6.

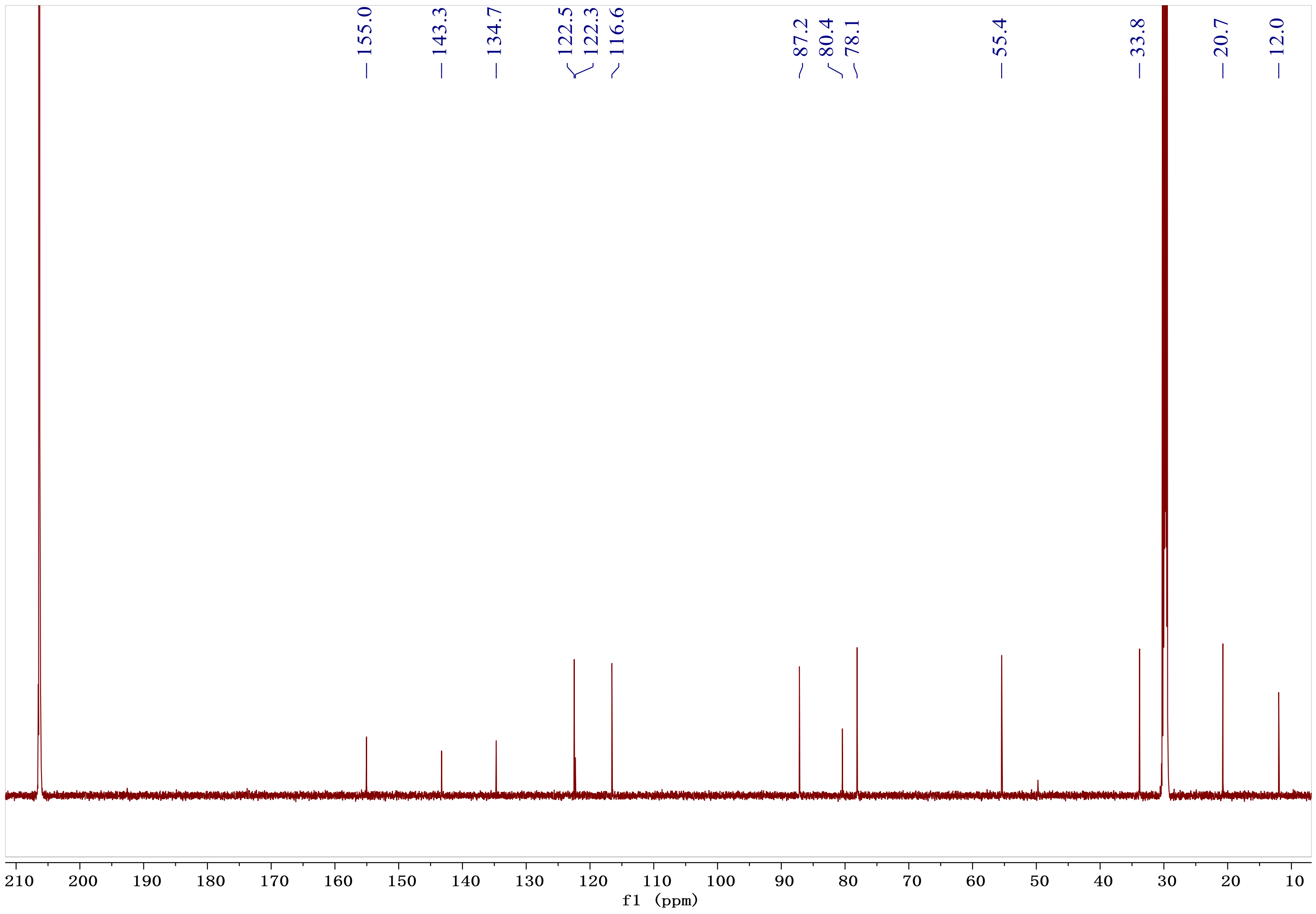

Figure S35. The [1]C NMR Spectrum of Compound **4** in Acetone-d_6.

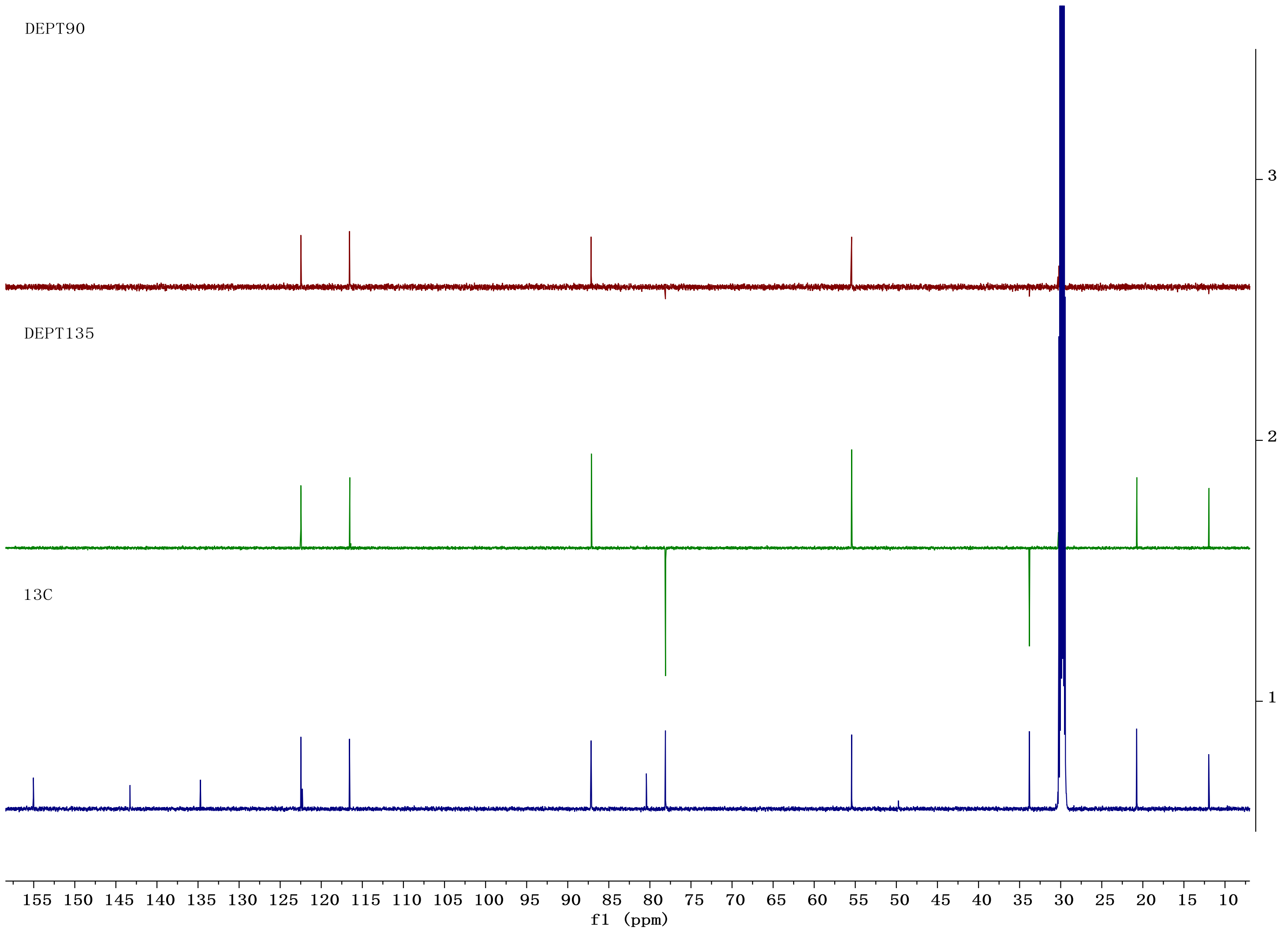

Figure S36. The DEPT Spectrum of Compound **4** in Acetone-d_6.

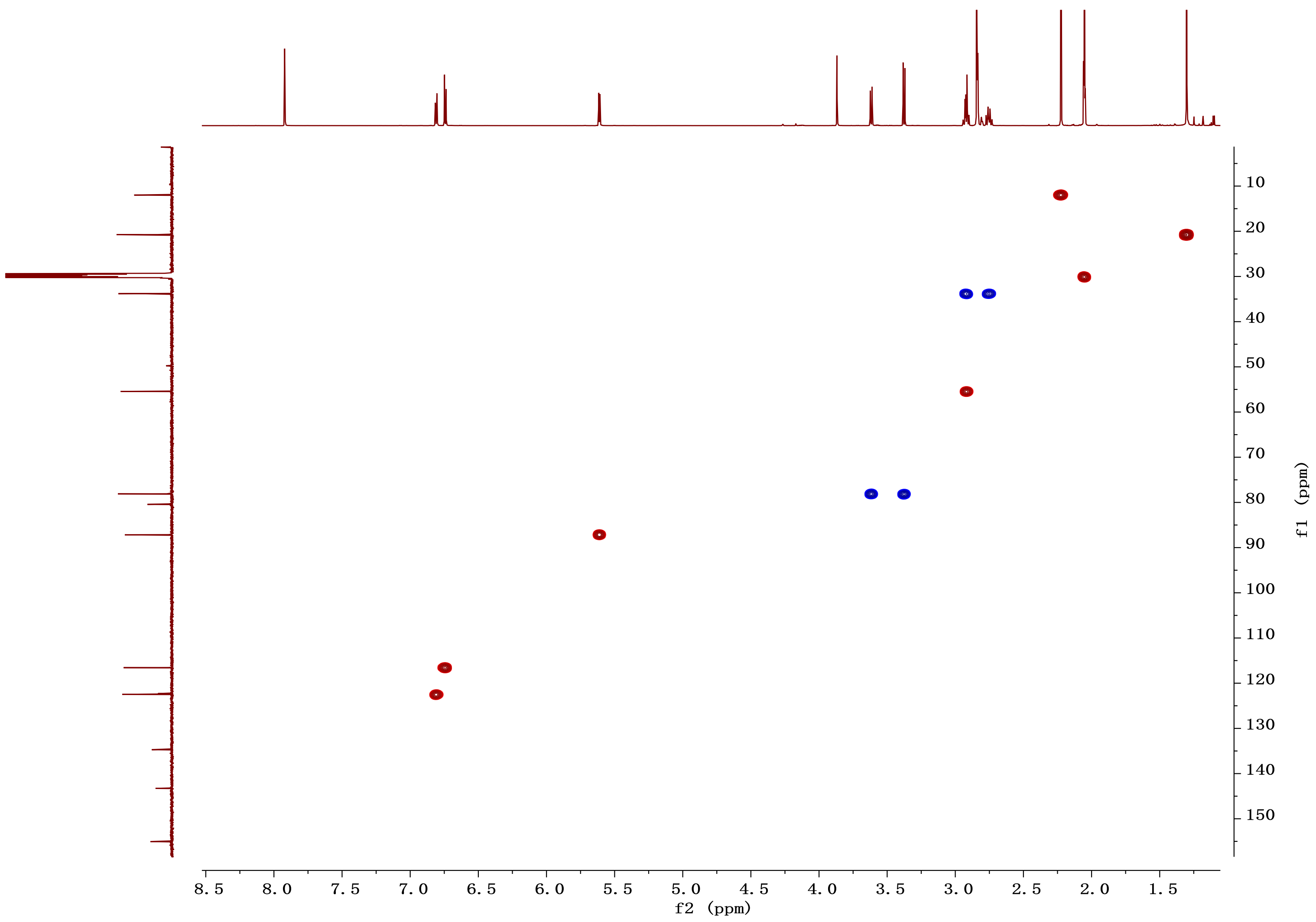

Figure S37. The edit-HSQC Spectrum of Compound **4** in Acetone-d_6.

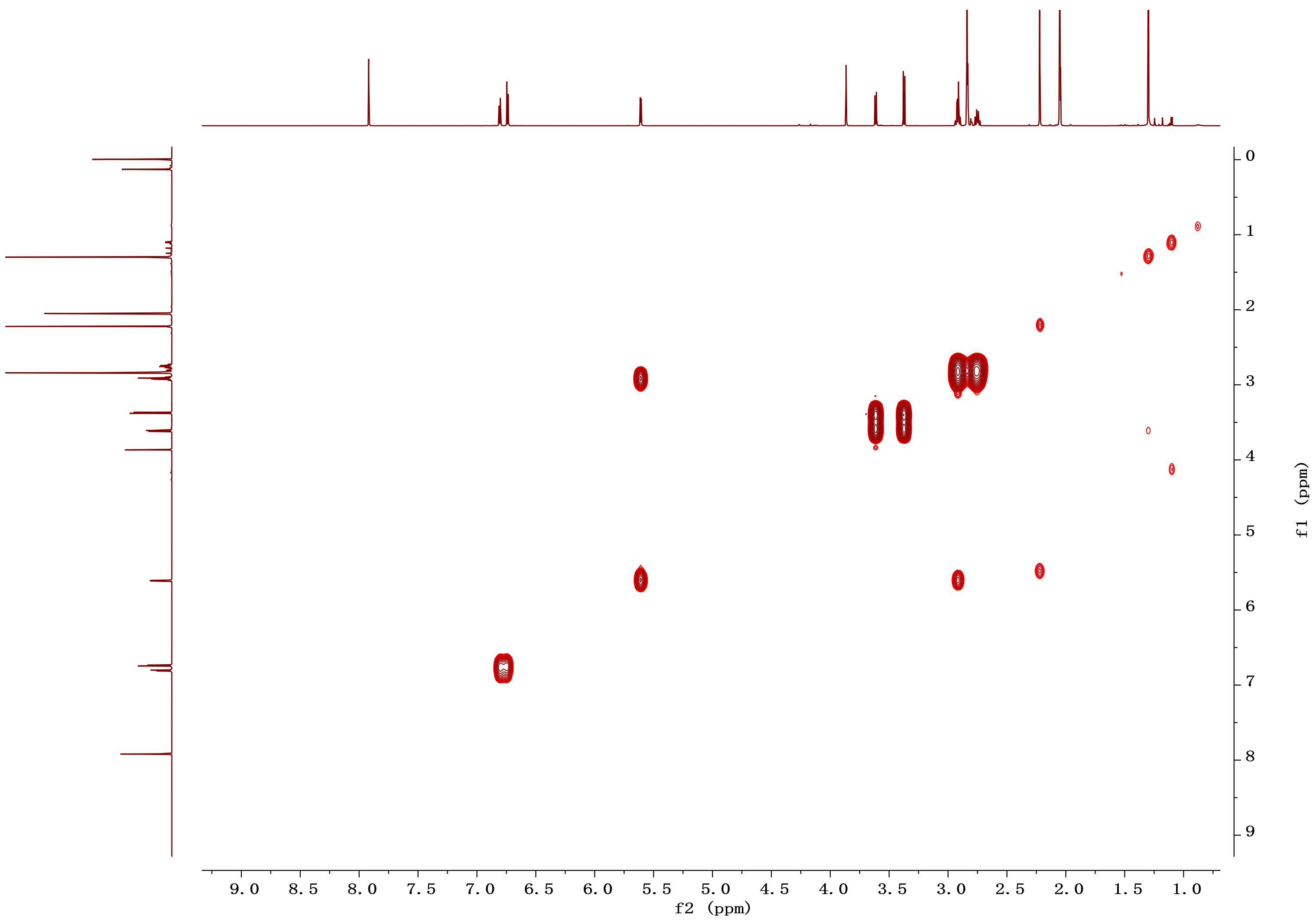

Figure S38. The ^{1}H-^{1}H COSY Spectrum of Compound **4** in Acetone-d_6.

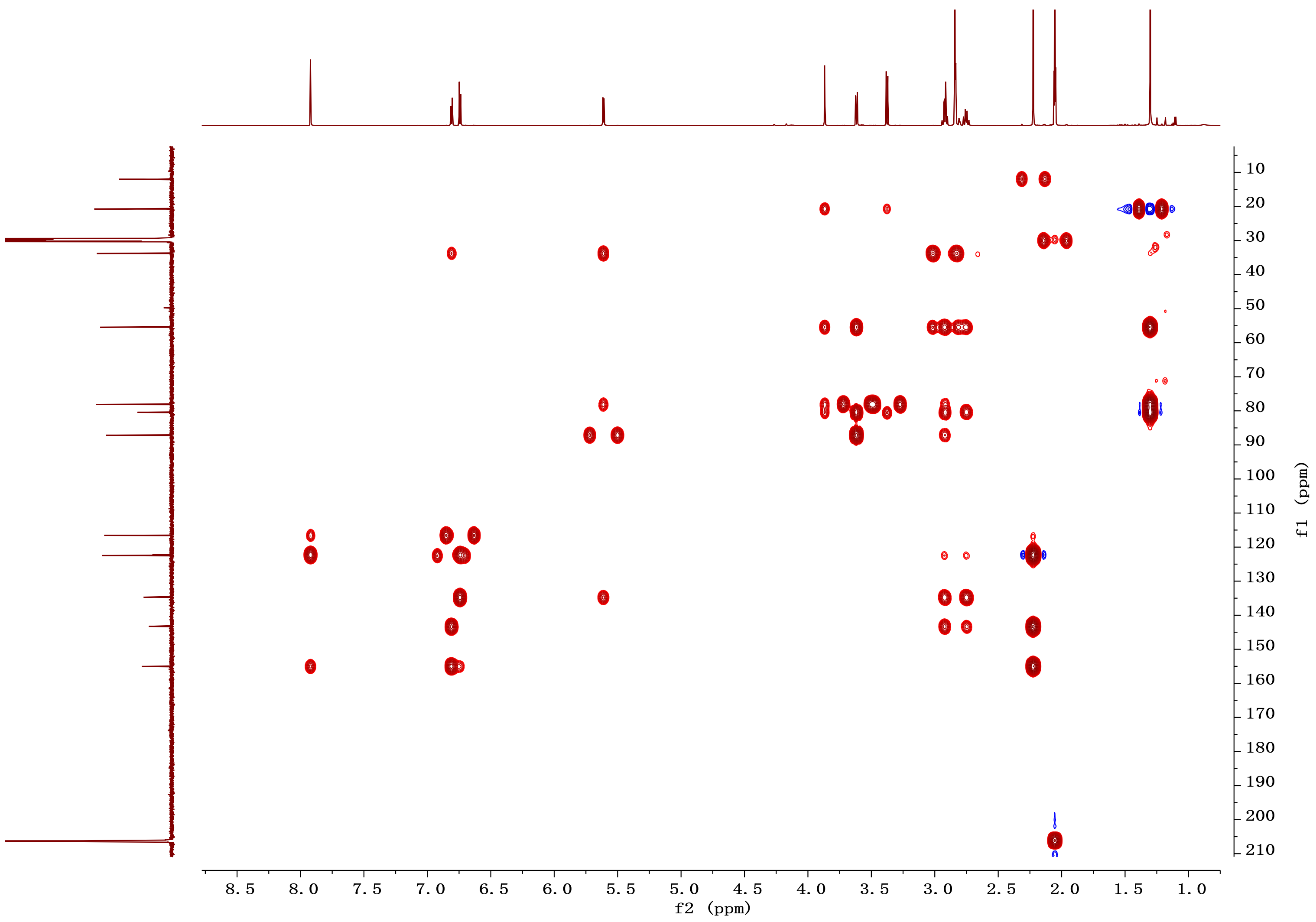

Figure S39. The HMBC Spectrum of Compound **4** in Acetone-d_6.

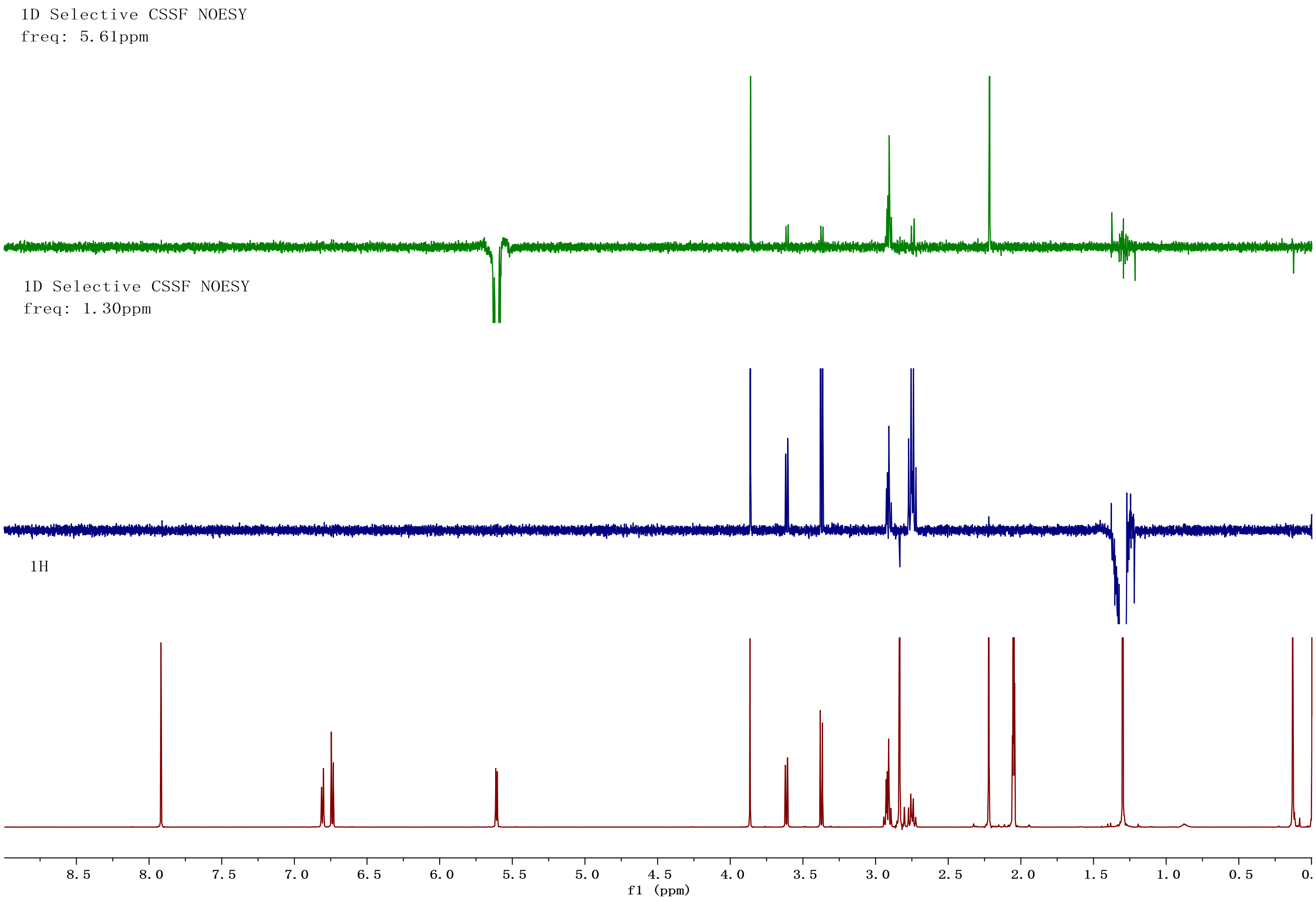

Figure S40. The 1D NOE Spectrum of Compound **4** in Acetone-d_6.
